# SIRT6 promotes mitochondrial fission and subsequent cellular invasion in ovarian cancer

**DOI:** 10.1002/2211-5463.13452

**Published:** 2022-06-24

**Authors:** Shreya Bandopadhyay, Parash Prasad, Upasana Ray, Damayanti Das Ghosh, Sib Sankar Roy

**Affiliations:** ^1^ Cell Biology and Physiology Division CSIR‐Indian Institute of Chemical Biology Kolkata India; ^2^ Department of Laboratory Medicine and Pathology Mayo Clinic Rochester MN USA; ^3^ Basic and Translational Research Saroj Gupta Cancer Centre and Research Institute Kolkata India

**Keywords:** actin polymerization, aerobic glycolysis, mitochondrial fragmentation, SIRT6

## Abstract

Ovarian cancer ranks fifth in terms of cancer mortality in women due to lack of early diagnosis and poor clinical management. Characteristics like high cellular proliferation, EMT and metabolic alterations contribute to oncogenicity. Cancer, being a “metabolic disorder,” is governed by various key regulatory factors like metabolic enzymes, oncogenes, and tumor suppressors. Sirtuins (SIRT1‐SIRT7) belong to the group of NAD^+^ deacetylase and ADP‐ribosylation enzymes that function as NAD^+^ sensors and metabolic regulators. Among sirtuin orthologs, SIRT6 emerges as an important oncogenic player, although its possible mechanistic involvement in ovarian cancer advancement is still elusive. Our data indicated a higher expression of SIRT6 in ovarian cancer tissues compared with the non‐malignant ovarian tissue. Further, we observed that overexpression of SIRT6 enhances glycolysis and oxidative phosphorylation in ovarian cancer cells. The energy derived from these processes facilitates migration and invasion through invadopodia formation by reorganization of actin fibers. Mechanistically, SIRT6 has been shown to promote ERK1/2‐driven activatory phosphorylation of DRP1 at serine‐616, which has an obligatory role in inducing mitochondrial fission. These fragmented mitochondria facilitate cell movement important for metastases. siRNA‐mediated downregulation of SIRT6 was found to decrease cellular invasion through compromised mitochondrial fragmentation and subsequent reduction in stress fiber formation in ovarian cancer cells. Thus, the present report establishes the impact of SIRT6 in the regulation of morphological and functional aspects of mitochondria that modulates invasion in ovarian cancer cells.

## Graphical abstract

SIRT6 is highly expressed in ovarian cancer cells and regulates tumor cell invasion. SIRT6 enhances the glycolytic rate and oxidative phosphorylation in ovarian cancer cells. SIRT6 induces phospho‐ERK1/2 mediated DRP1 phosphorylation at serine‐616 to facilitate mitochondrial fission. Fragmented mitochondria translocate in an anterograde manner along with actin fibers to facilitate invadopodia formation and associated cancer cell movement.

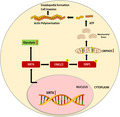

**Abbreviations**
DAPI 4′: 6‐diamidino‐2‐phenylindole
DRP1: dynamin‐related protein 1
ECAR: extracellular acidification rate
HIF: hypoxia‐inducible factor
MAPK: mitogen‐activated protein kinase
MMP: matrix metalloprotease
NAD: nicotinamide adenine dinucleotide
OCR: oxygen consumption rate
siRNA: short interfering RNA
SIRT6: Sirtuin 6

Ovarian cancer is a deadly gynecological malignancy owing to late diagnosis and poor prognosis [1]. Characteristic rapid metastasis and the dearth of early screening measures pose this cancer type as a severe clinical challenge. To sustain their existence inside the host body, cancer cells rewire their cellular metabolism and bioenergetic phenotypes [2]. Warburg effect stands as a vital metabolic adaptation wherein cancer cells undergo rapid aerobic glycolysis. This is marked by the surge in glucose consumption and lactate secretion with adequate ATP production [3, 4]. Additional ATP procurement from various cellular metabolic pathways is implemented by mitochondria through oxidative phosphorylation (OXPHOS) [5, 6]. Cancer cells need to attain metabolic plasticity that confers a hybrid metabolic phenotype equilibrating both glycolysis and OXPHOS. Aerobic glycolysis or Warburg Effect is the metabolic adaptation by which proliferating tumor cells accumulate the biomass necessary to pursue the associated proliferative state [7, 8, 9]. Epithelial‐mesenchymal transition (EMT) necessitates this precise metabolic state to fuel the establishment of tumor cells in secondary sites. Mechanistically, invasion from the primary tumor site is driven by distinct cytoskeletal rearrangements facilitated by actin polymerization at the leading edge of the migratory cells. Incessant cycles of mitochondrial fission and fusion to reinforce cell migration are attributed to the heterogeneous distribution of this dynamic organelle [10]. Dynamin‐related protein 1 (DRP1) is a critical mitochondrial fission protein that induces mitochondrial fragmentation [11]. Increased fission along with higher OXPHOS propels tumor growth and metastases. This is since heightened fission promotes the ascending number of fragmented mitochondria at the leading edges, a prerequisite for cell motility [12, 13].

Sirtuins are a group of NAD^+^ dependent histone deacetylases and ADP‐ribosylases discovered as “Yeast Sir2,” possessing diverse functions in all groups of organisms [14, 15, 16]. Among them, Sirtuin‐6 (SIRT6) has emerged as a crucial member with regulatory roles in aging, metabolism, DNA damage repair, lifespan determination, fat mobilization, stress response, apoptosis, autophagy, and cancer [17, 18, 19]. The action of SIRT6 is reported to be tissue‐specific, and thus, it acts as a tumor suppressor in some cancer types, while, as an oncogene in others. SIRT6 is found to be upregulated in hepatocellular carcinoma, breast cancer, ovarian cancer, esophageal cancer, lung cancer, osteosarcoma, papillary thyroid cancer, and prostate cancer [20, 21, 22, 23, 24, 25]. The proliferative effects of SIRT6 have been explored in breast cancer, melanoma, and esophageal cancers [21, 26, 27]. SIRT6 activates extracellular signal‐regulated kinase 1/2 (ERK1/2) in non‐small cell lung carcinoma and osteosarcoma to drive metastatic spread via MMP9 upregulation [20, 28, 29]. Again, ERK1/2 regulates mitochondrial function, cell invasion, cell proliferation, and glycolysis in cancer cells [30, 31]. High glycolytic rate releases lactate as the end‐product, which causes acidification in the tumor and stromal cells [32]. This lower pH is responsible for the cellular matrix degradation through upregulation of MMP activity, facilitating secondary site metastases [33]. Therefore, the advancement of tumor growth by increased migration and invasion calls for the rewiring of cellular metabolism. SIRT6 being a “metabolic sensor” fosters metabolic changes conducive to tumor growth [34]. As tumor cells need to reprogram their metabolism continuously through different stages of cancer progression, we focused on the metabolic alterations manifested by SIRT6. At the same time, enhanced mitochondrial fragmentation and their localization to the peripheral leading edge of migratory cells provide ATP to support continuous dynamics of the actin cytoskeleton and buffer the Ca^2+^ levels for the generation of new focal adhesion points [12, 35]. Likewise, few reports suggest a connection between mitochondrial function and cancer cell metastases eliciting stress fiber formation [5]. The inhibition of key mitochondrial fission regulators decreases mitochondrial movement velocities and orientation [36]. Considering this background information, we tried to explore the possible metabolic and bioenergetic alterations manifested by SIRT6 in promoting the invasiveness of cancer cells.

Ovarian cancer cells show increased metabolic alterations than non‐malignant ovarian cells [37]. This report highlights the importance of SIRT6 as an oncogenic driver across ovarian cancer tissues and cell lines. We observed that SIRT6 induces ERK1/2‐dependent mitochondrial fission, which, in turn, can regulate cancer cell invasion and migration. Therefore, our observations revealed an important role of SIRT6 in the regulation of mitochondrial morphology and how this facilitates ovarian cancer invasion.

## Materials and methods

### Cell culture and treatments

Human ascitic‐fluid‐derived ovarian cancer cell lines, PA1 (Cat no. CRL‐1572, ATCC, Manassas, VA, USA) and SKOV3 (Cat no. HTB‐77, ATCC), were cultured in Minimum Essential Medium‐alpha (Cat no. AL080A, Himedia, Mumbai, India) and RPMI‐1640 media (Cat no. AL199A, Himedia), respectively. Human immortalized ovarian surface epithelial cells, IOSE‐364 (a kind gift from C. Salamanca, Vancouver, BC, Canada), were maintained in MCDB‐105 (Cat no. M6395, Sigma‐Aldrich) and Medium‐199 (Cat no. 11150059, Gibco, Waltham, MA, USA) in a 1 : 1 ratio. Cell lines maintained in their respective medium were supplemented with 10% Fetal Bovine Serum (FBS; Gibco, Cat no. 16000‐044), 100 units·mL^−1^ penicillin and 100 μg·mL^−1^ streptomycin (Cat no. 15140163, Gibco) at humidified 37 °C with 5% CO_2_ incubation. Oligomycin‐A (Cat no. 75351, purchased from Sigma‐Aldrich) was used at an effective concentration of 500 nm [38], mdivi‐1 (Cat no. M0199, purchased from Sigma‐Aldrich) was used at 14 μm concentration, ERK inhibitor (Cat no. 328006) at 10 μm, 2‐deoxy‐d‐glucose (Sigma‐Aldrich, Cat no. D8375) at 1 mm, HIF1α inhibitor (Calbiochem, Cat no. 400089) and MMP9 inhibitor‐I (Cat no. 444278) at 10 nm concentration according to previous reports [39, 40], [41].

### Plasmid constructs, siRNAs, and transfections

SIRT6‐Flag expression plasmid, a gift from Eric Verdin (Cat no. 13817, Addgene, Watertown, MA, USA) constructed into pcDNA 3.1 (+) vector (Invitrogen, Waltham, MA, USA), was used for overexpression studies. In all the experiments, pcDNA 3.1 (+) (empty vector/EV) transfected cells were considered as control [22]. For SIRT6 overexpression studies, 1 μg plasmid construct was transfected at 1 × 10^6^ cells per well in 6‐well plates and at 2 μg for 2 × 10^6^ cells per well in 60‐mm cell culture dishes. Lipofectamine‐2000 (Cat no. 11668019, Invitrogen) was used for transfection experiments in OPTI‐MEM (cat no. 11058021, Gibco) medium following the manufacturer's instructions. 4‐h post‐transfection, OPTI‐MEM was changed to complete serum‐containing growth medium. After transfection, the cells were harvested for different experiments for 24 h.

For knockdown studies, Human SIRT6 siRNA (Cat no. sc‐63028, Santa Cruz Biotechnology, Dallas, TX, USA) was transfected against scrambled siRNA (Cat no. sc‐37007, Santa Cruz Biotechnology) as the control in ovarian cancer cells, at 10 nm concentration using Lipofectamine RNAiMAX (Cat no. 13778150, Invitrogen) and incubated for 48 h.

### RNA isolation and quantitative Real‐Time PCR (qPCR)

Total cellular RNAs were isolated with TAKARA RNA‐iso Reagent (Cat no. 9109 Takarabio, Kyoto, Japan) using the manufacturer's instructions. After checking the RNA integrity by running agarose gel, first‐strand cDNA was synthesized from 500 ng of isolated RNAs, using the iscript™ cDNA Synthesis kit (Cat no. 1708890; Bio‐Rad, Hercules, CA, USA). This was followed by Quantitative Real‐time PCR (qPCR) analysis using iTaq Universal SYBR green supermix (Cat no. 1725120, Bio‐Rad) and ABI 7500 Fast Real‐Time PCR system (Applied Biosystems, Waltham, MA, USA). Melt curve analysis was performed followed by the qPCR. The comparative *C*_T_ (ΔΔ*C*_T_) method was used to calculate relative gene expression levels after normalization with 18S rRNA (housekeeping gene). Fold change of target mRNA was calculated by $\Delta C_{T}=2^{\left(\Delta C_{T}\left(\text{treatment}\right)\right)-\Delta C_{T}\left(\text{Control}\right))}$ formula, where ΔC_*T*_ is the C_*T*_ of the target gene subtracted from the C_*T*_ of 18S rRNA. Fold change in expression is designated in the *Y*‐axis (mean ± SEM). All the primers have been designed with primer express 3.0 software (Applied Biosystems) and verified with Primer‐BLAST. The primers were procured from Integrated DNA Technology (IDT) with their sequences displayed in Table 1.

**Table 1:** Primer sequences used for mRNA abundance analysis.

Gene name	Forward primer (5′‐3)	Reverse primer (5′‐3)	Tm (°C)
18s rRNA	GATTCCGTGGGTGGTGGTGC	AAGAAGTTGGGGGACGCCGA	59
SIRT1	CGGGAATCCAAAGGATAATTCA	CCTCGTACAGCTTCACAGTCAACT	59
SIRT2	GCTGGAACAGGAGGACTTGGT	TGGCGCTGACGCAGTGT	59
SIRT3	CCCGAGGCCATTTTTGAA	CTCCTTGGCCAAAGTGAAAAAG	59
SIRT4	CCCATCCAGCATGGTGATTT	CTACGAAGTTTCTCGCCCAGTAC	59
SIRT5	CATCACCCAGAACATCGATGAG	GCTACCATGGATCTCCAGAAGGT	59
SIRT6	GCACGACCGCCATGCT	GCTTCATGAGCCGGGTCAT	59
SIRT7	GGCAGGATCCCATTTTCTCA	GACTGTGGCTGCCTTCTTCAC	59
MMP9	ACCTCGAACTTTGACAGCGAC	GAGGAATGATCTAAGCCCAGC	59
MMP2	TGATCTTGACCAGAATACCATCGA	GGCTTGCGAGGGAAGAAGTT	59
HIF1α	TGAACATAAAGTCTGCAACATGGA	TGAGGTTGGTTACTGTTGGTATCATATA	59

### RNA transcriptomics analysis

mRNA was isolated according to the mentioned protocol. Global transcriptomics analysis was carried out with Bionivid Technology Pvt. Ltd, Bangalore, India, in PA1 cells transfected with EV and pcDNA 3.1‐SIRT6 plasmid for the experiment (Fig. 2). The transcriptomics data have been submitted to the NCBI SRA portal with BioProject ID PRJNA783744. The data analysis of the transcriptome data was carried out using the Tuxedo pipeline. cDNA prepared from mRNA samples was checked for quality and integrity followed by library construction. Cluster generation was carried out, and samples were run on next‐generation sequencing using Illumina SBS Technology. With the use of Fastq files, the sequencing reads were mapped to Hg38 UCSC as a Human Reference genome using tophat (v. 2.1.1) [42]. CUFFLINKS‐based probabilistic model (cufflinks package v2.2.1) for paired‐end sequencing was utilized to estimate the expression level and assemble transcripts based on gene annotation from NCBI RefSeq [43]. Cuffmerge was then applied to merge transcripts from individual samples for producing a unified transcriptome file. For the gene expression analysis, estimated expression levels were converted from FPKM units (number of reads × 10^9^/transcript length/library). CuffDiff protocol was used to identify differentially expressed transcripts. A fold change of more than two having a *P*‐value of ≤ 0.05 was used as a cutoff for the identification of differentially expressed genes. david bioinformatics resources V.6.7 [44,45] was used for functional annotation, and unsupervised hierarchical clustering was carried out with cluster 3.0 [46]. Visualization of heat maps was done using Java Treeview, and Volcano plots were generated for differential gene expression.

### Western blot

Following cell transfection, the cells from 60‐mm tissue culture dishes were collected in cold PBS after centrifugation at 800 ***g*** for 5 min and thereafter were processed using the standard protocol [47]. The cell pellet was resuspended in modified RIPA lysis buffer (50 mm Tris pH 8, 150 mm NaCl, 2 mm EDTA, 0.1% SDS, 1% NP‐40) supplemented with PMSF, DTT and 1X Protease‐inhibitor cocktail (Pierce, Thermo Fisher Scientific, Waltham, MA, USA) and incubated in ice for 20 min. Cells were sonicated 5 times for 30 s each at amplitude 50 with 10‐s intervals using bath Sonicator (Labman Scientific Instruments, Tamil Nadu, India). Total cell protein was then obtained by centrifuging the cells at 10 000 ***g*** for 10 min at 4 °C, followed by concentration measurement using the Bronsted–Lowry method. After loading equal concentrations of proteins (100 μg), they were resolved and separated using 7%, 10% and 12% SDS‐Polyacrylamide gel electrophoresis depending upon the protein sizes. Gels were transferred to polyvinylidene fluoride membrane (PVDF) for timespans depending upon the size of the proteins. Non‐specific binding to the membrane was blocked by incubation with 3% Bovine Serum Albumin (Himedia) in TBST [20 mm Tris–HCl (pH 7.4), 150 mm NaCl and 0.1% Tween 20] solution. Overnight incubation at 4 °C with respective primary antibodies and for 2 h with secondary horseradish peroxidase (HRP)‐conjugated antibodies (compatible for chemiluminescence) at room temperature was carried out. Antibodies used for western blots are listed in Table 2. Full blot images of all western blots have been provided in Figs S4–S6.

**Table 2:** Antibody details used for western blot analysis.

Sl. No.	Target	Company	Catalog no.
1	SIRT6	Cell Signaling Technology	12486S
2	α‐Tubulin (11H10)	Cell Signaling Technology	2125S
3	PCNA	Cell Signaling Technology	2586S
4	Ki‐67 (8D5)	Cell Signaling Technology	9449S
5	Phospho‐DRP1 (ser‐616)	Cell Signaling Technology	3455BC
6	DRP1 (D6C7)	Cell Signaling Technology	8570S
7	MFN1 (D6E2S)	Cell Signaling Technology	14739BC
8	MFN2	ABclonal	A12771
9	Anti‐mouse IgG‐HRP	Cell Signaling Technology	7076
10	Anti‐rabbit IgG‐HRP	Cell Signaling Technology	7074
11	p‐p44/42 MAPK (Erk1/2) (thr202/tyr204)	Cell Signaling Technology	9101
12	p44/42 MAPK (Erk1/2)	Cell Signaling Technology	9102
13	Goat anti‐rabbit AF488	Invitrogen	A11008

### Confocal microscopy

For mitochondrial conformation analysis, 1 × 10^6^ cells plated on the coverslips were transfected for both overexpression (24 h) and RNA interference (48 h) studies. Mitotracker Red (Cat no. 7512, Invitrogen) at a concentration of 50 nm was added to the cells in serum‐containing media and incubated for 30 min at 37 °C. Cells were then fixed with 4% paraformaldehyde for 15 min and permeabilized with 0.1% Triton X‐100 for 15 min. Cells were then incubated in AF488‐tagged‐Phalloidin Green (Cat no. A32731, Invitrogen) for 20 min. For antibody staining, p‐DRP1^ser616^, MFN1, and MFN2 were added in 1 : 200 ratio and incubated overnight, followed by secondary antibody addition on the next day for 2 h. Staining of the nucleus with 4′,6‐diamidino‐2‐phenylindole or DAPI (Cat no. 18668, SRL, 0.25 μg·mL^−1^) was done for 5 min. The images were acquired by Leica confocal microscope (TCS SP8, Buffalo Grove, IL, USA) with 63× oil immersion objective lens. Analysis of mitochondrial length was performed using lasx software [39]. Line‐scan analysis between AF488‐tagged‐phalloidin and Mitotracker Red was carried out using imagej software [39, 48].

### Invasion assay

Matrigel‐coated 24‐well chamber (Cat no. 354480, Corning‐BD, New York, NY, USA) inserts were used for the invasion studies following the manufacturer's protocol. Briefly, 5 × 10^4^ cells·mL^−1^ were kept in the chamber insert with 0.1 mL of incomplete media having 0.5 mL of complete media beneath the chamber insert in each well. The cells were allowed to invade for 22 h and were followed by methanol fixation and crystal violet staining. After drying, the Matrigel insert was removed from the chamber and mounted on a slide. The cells were visualized under light microscope (Evos‐Invitrogen) with 20× objective. From each image, a random cell count was performed, averaged, and expressed in a bar diagram.

### Migration assay

1 × 10^6^ cells were plated in 6‐well plates for this experiment. After they attained 60% confluency, they were transfected and/or treated with the inhibitors as mentioned earlier in the materials and methods section. Cell growing till 100% confluency was scratched with a 200 μL tip head and allowed to heal. At 24 h, images of the wound were taken in phase contrast mode with EVOS‐FL microscope (Invitrogen) with 10× objective. The wound width was measured in imagej Software [41].

### Extracellular flux analysis

5 × 10^4^ cells were seeded in 100 μL of complete cell culture medium in a 24‐well plate (Agilent, Santa Clara, CA, USA) and kept to rest for 4 h at 37 °C incubator for adherence. Then, 150 μL of the complete medium was added to each well to bring the total volume in each well to 250 μL. This was followed by incubation of cells for 16 h followed by transfection with EV, Scrambled siRNA, pcDNA 3.1‐SIRT6 plasmid and SIRT6 siRNA in specific wells in triplicate. 24‐h post‐transfection, glyco‐stress and mito‐stress assays were performed for overexpression studies and 48‐h incubation protocol was followed for knockdown studies in case of siRNAs.

For Glycolysis stress assay medium preparation, 2 mm glutamine and 2 mm sodium pyruvate were added into XF assay medium, pH 7.4. The cells transfected previously on 24‐well tissue culture plate were washed twice with this assay medium and incubated at 37 °C for 1 h before the assay. Glyco‐stress assay (Agilent, Cat no. 103344‐100) test compounds 10 mm glucose, 1 μm oligomycin‐A and 2.5 mm 2DG were injected into the Utilization plate through appropriate ports of the cartridge (XFe‐24 Extracellular Flux Analyzer). The assay was run following the standard protocol of this instrument as available in their website (https://www.agilent.com/cs/library/usermanuals/public/XF_Glycolysis_Stress_Test_Kit_User_Guide.pdf).

Similarly, for Mito Stress Assay (Agilent, Cat no. 103015–100) the Mito‐stress assay medium was prepared by adding 2 mm glutamine, 25 mm glucose, and 2 mm sodium pyruvate into XF assay medium, pH 7.4. Transfected cells grown on the culture plate were washed twice with this assay medium and kept in the same medium in 37 °C incubator for 1 h before the assay. Inhibitors including 1 μm Oligomycin‐A, 0.9 μm FCCP and 1 μm Antimycin‐A/Rotenone were injected into the appropriate ports during this assay as per the protocol of the instrument (https://www.agilent.com/cs/library/usermanuals/public/XF_Cell_Mito_Stress_Test_Kit_User_Guide.pdf).

### Fluorescence‐based immunohistochemistry

Paraffin‐embedded tissue blocks were procured from Saroj Gupta Cancer Centre and Research Institute, Kolkata, with proper ethical clearance from the Institutional Ethics Committee with approval number—IEC SGCCRI REF NO. 16/2/2018/Non‐Reg/SSR/3. Informed written consent from all the patients was taken according to the 1964 Helsinki declaration, and information regarding the nature and stage of all the tissues was verified by the clinicians and pathologists of this institution. Tissue processing by ethanol gradation followed by fixation on slides was carried out at optimum temperatures. Then, deparaffinization using percentage gradients of ethanol was done. Further, antigen retrieval using sodium citrate for 20 min at 100 °C was performed, followed by blocking in 3% bovine serum albumin (BSA) solution in PBS. Primary antibody of Rabbit anti‐SIRT6 (1 : 1000) incubation was carried out overnight at 4 °C, and then the slides were kept with secondary antibody Alexa fluor‐488‐anti‐rabbit for 2 h. The cell nucleus staining was done with DAPI for 5 min. Images were captured using Leica confocal microscope (TCS SP8).

### Hematoxylin and eosin staining

The tissue slides were stained with hematoxylin and eosin using the standard protocol, and images were taken in bright‐field microscope (LM‐52‐1705, Lawrence and Mayo, Mumbai, India).

### Procurement of patient survival plot

The Kaplan–Meier plot for SIRT6 expression in ovarian cancer patients was acquired from http://www.oncolnc.org as mentioned in our previous report [41], with the total number of patients = 73, in each category. The lower percentile and upper percentile both were taken as 25, and the plot acquired had a *P*‐value of 0.563.

(http://www.oncolnc.org/kaplan/?lower=25&upper=25&cancer=OV&gene_id=51548&raw=sirt6&species=mRNA)

### Acquisition of Human Protein Atlas data

The archival SIRT6 protein expression analysis images in different ovarian cancer patients along with normal tissues were acquired from the publicly available repository in protein atlas (https://www.proteinatlas.org/ENSG00000077463‐SIRT6/pathology/ovarian+cancer#ihc).

### Statistical analysis

All the experiments were performed in three biological replicates with their statistical analysis being done in graphpad prism‐5 (San Diego, CA, USA). The data were graphically represented as Mean ± SEM. In the case of paired samples, two‐tailed Student's *t*‐test was performed and *P*‐value was generated. Statistical evaluation of higher number of samples was done by one‐way ANOVA followed by Bonferroni's Multiple Comparison Test. The level of significance is calculated as α = 0.05 (95% confidence interval). Data Information: ns—non‐significant, *P‐value < 0.05, **P‐value < 0.01, and ***P‐value < 0.001.

## Results

### SIRT6 increases ovarian cancer cell invasion

**Fig. 1:**
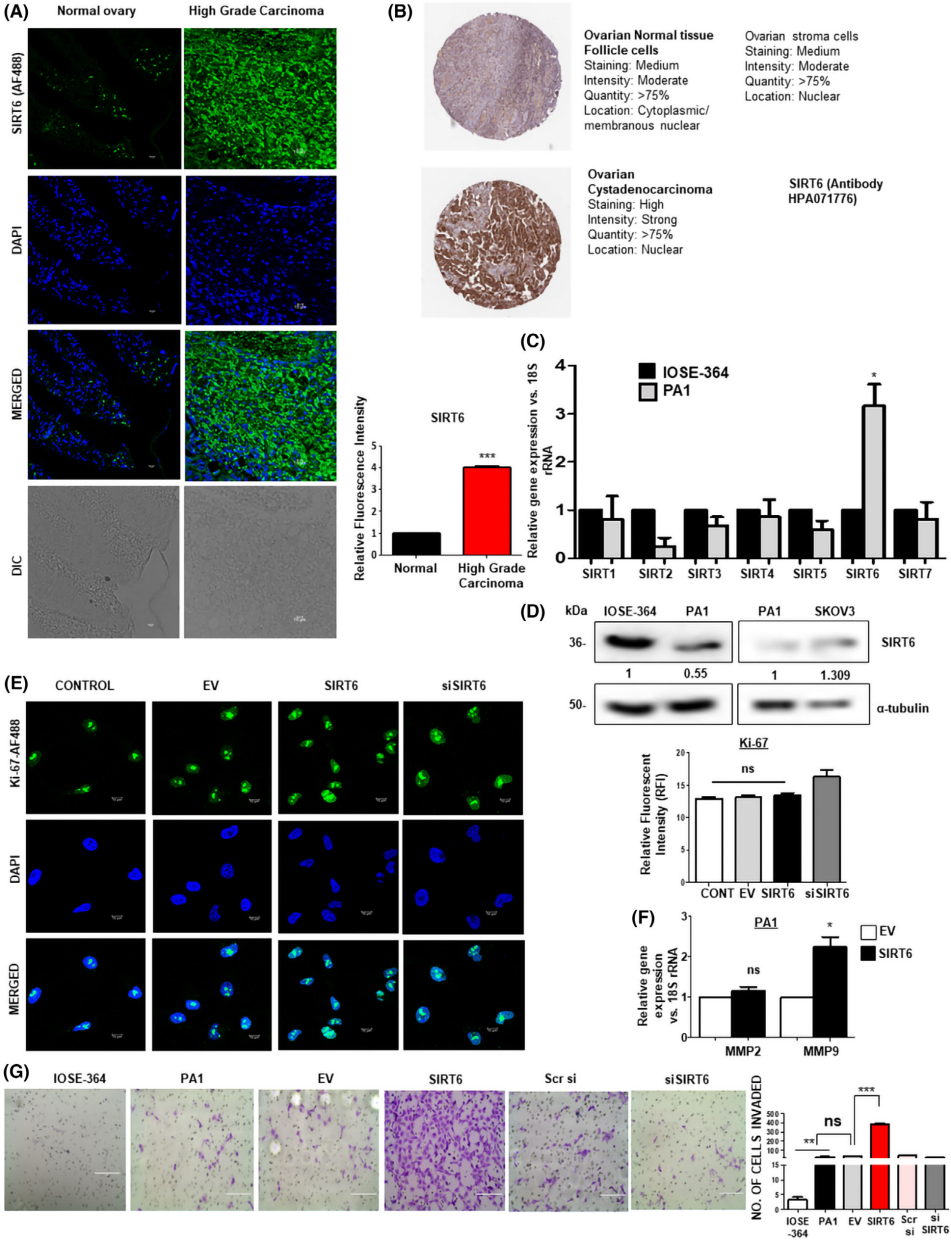
SIRT6 protein expression is high in ovarian cancer, which regulates the metastatic phenotype (a) Immunohistochemical staining shows high SIRT6 levels in high‐grade carcinoma patient tissue sections with corresponding bar diagram for expression levels. Scale bar = 10 μm. *n* = 3. Paired two‐tailed *t*‐test was done to calculate the *P*‐value. (B) Human Protein Atlas data show high SIRT6 expression in ovarian cancer tissue. (C) qPCR data show elevated SIRT6 levels in PA1 cell lines compared with IOSE‐364. *n* = 3, Paired two‐tailed *t*‐test was done to calculate the *P*‐value. (D) Endogenous SIRT6 expression in IOSE‐364 *vs*. PA1 and PA1 *vs*. SKOV3 cell lines. (E) Ki‐67 staining in control (untransfected), EV‐transfected, SIRT6‐overexpressed, and SIRT6 siRNA transfected PA1 cells and relative fluorescence intensity have been depicted as bar graphs. Scale bar = 10 μm, *n* = 3 (F) qPCR data showed non‐significant change in MMP2 and an increase in MMP9 in EV‐transfected *vs*. pcDNA 3.1‐SIRT6‐transfected PA1 cells. *n* = 3, Paired two‐tailed *t*‐test was done to calculate the *P*‐value. (G) Matrigel invasion studies show the number of invaded cells in IOSE‐364, untransfected PA1 (control), EV‐transfected, SIRT6‐overexpressed, scrambled siRNA, and SIRT6‐silenced PA1 cells. Corresponding bar diagram to show the number of invaded cells per field. Scale bar = 200 μm. *n* = 3, ANOVA was done to calculate the *P*‐value. Error bars represent standard error of mean (SEM) from three independent experiments. ns—non‐significant, *P‐value < 0.05, **P‐value < 0.01, ***P‐value < 0.001. [Colour figure can be viewed at wileyonlinelibrary.com]

To decipher the biological significance of SIRT6 in ovarian cancer, we checked the expression of SIRT6 in patients' tissue sections and found a significant increase of SIRT6 in high‐grade serous ovarian cancer tissues (HGSOC) compared to that in non‐cancer ovarian sections (Fig. 1A). H&E staining was performed to confirm the histopathology of the tissues (Fig. S1A). Human protein atlas data also revealed significantly enhanced expression of SIRT6 in ovarian cystadenocarcinoma with strong intensity and nuclear localization (Fig. 1B). However, we did not find any significant relationship between SIRT6 mRNA expression and ovarian cancer patient survival probability, which reflects that the levels of SIRT6 proteins do not match with mRNA expression (Fig. S1B). Expression profiling of Sirtuins was performed in normal ovarian epithelial IOSE‐364 *vs*. PA1 ovarian cancer cell lines. The qPCR analysis confirmed a significant increase in the SIRT6 mRNA expression in PA1 cells as compared to IOSE‐364 cells (Fig. 1C). Initially, the endogenous protein expression level of SIRT6 in IOSE‐364 and PA1 was studied which depicted a slight decrease in SIRT6 level in PA1 cell line, so we performed SIRT6 overexpression in PA1 for most of our experiments. This was done to understand the effect of increased SIRT6 in PA1 ovarian cancer cell line instead of comparing the cancer cells directly to the IOSE‐364 cells. However, SKOV3 cell line showed increased SIRT6 levels as compared to PA1, which makes this cell line useful for both SIRT6 overexpression and SIRT6 siRNA‐mediated knockdown studies (Fig. 1D). After checking the efficiency of SIRT6 plasmid and SIRT6 siRNA in PA1 cell line, we proceeded with the investigations (Fig. S1C,D). To study the effect of SIRT6 in cancer cell proliferation, the expression of Ki‐67 proliferation marker was assessed in ovarian cancer cells. No significant change in Ki‐67 levels was found in SIRT6‐transfected PA1 cells (Fig. 1E). Another basic feature of cancer cells is invasion, which is responsible for the metastasis of tumor cells to the secondary sites, where they form new tumors. They undergo epithelial‐to‐mesenchymal transition (EMT) to gain migratory capabilities and invade through the basement membrane [49]. The tumor cells secrete matrix metalloproteases (MMPs), like MMP2 and MMP9, which helps to degrade the collagen matrix to make a path for invading cells. qPCR data showed an increased expression of MMP9 but no change in MMP2 levels on SIRT6 overexpression (Fig. 1F). Matrigel invasion studies revealed a significant increase in cell numbers of PA1 and SKOV3 invading through the matrigel membrane, whereas silencing SIRT6 with siRNA reduced the cell invasion (Fig. 1G, Fig. S1E). Therefore, increased SIRT6 expression has been found to promote the invasiveness of ovarian cancer cells.

### Gene regulatory network revealed the tumor modulatory directives of SIRT6

**Fig. 2:**
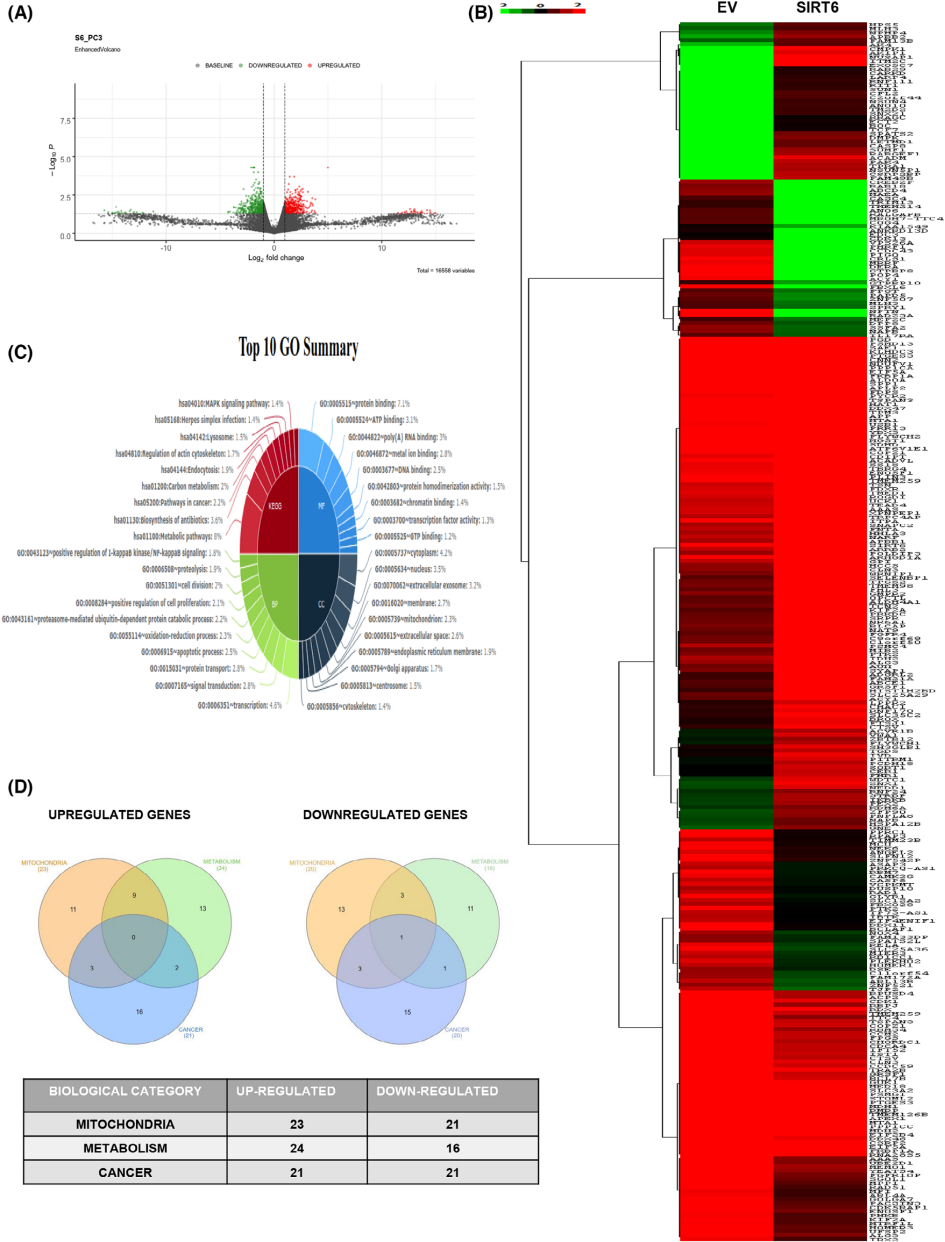
Transcriptomics data indicate the tumorigenic role of SIRT6. (A) Volcano plot depicting a total of 16 558 genes among which 16256 genes were at the baseline level, 161 showed upregulation (red dots) and 141 genes were downregulated (green dots). (B) Heat map was generated according to −log_10_P values with lower ‐log_10_P having high significance levels. (C) Data obtained were grouped into different GO pathways. (D) Venn diagrams were produced considering 3 heads with maximum genes involved that are altered by SIRT6 *viz*. mitochondria, metabolism, and cancer. [Colour figure can be viewed at wileyonlinelibrary.com]

To further extrapolate how SIRT6 regulates cancer progression, global transcriptomics analysis (BioProject ID PRJNA783744) was performed with mRNAs isolated from PA1 cells transfected with empty vector (control) and pcDNA 3.1‐SIRT6 plasmid. The Volcano plot (Fig. 2A) was generated to extract a total of 16 558 variables, among which 16 256 genes had baseline expression levels, 161 genes were upregulated (shown as red dots), and 141 were downregulated (shown as green dots). The genes that were considered to have lower −log_10_P values with higher significance and higher log_2_‐fold change were categorized in the heat map produced by Java Tree view (Fig. 2B). The data obtained were grouped into different GO pathways (Fig. 2C), and considering the pathways with maximum hits, Venn diagrams were plotted. The highest number of target genes were found to be involved in the regulation of mitochondrion, metabolic pathways and pathways in cancer. The differentially expressed genes were segregated into two Venn diagrams of upregulated and downregulated genes (Fig. 2D). Therefore, this representation shows the coordination of cancer‐pathway‐related genes with the players of mitochondrial function and metabolic regulation, predicting their importance in SIRT6‐induced cancer cell modulation.

### SIRT6 regulates glycolysis in tumor cells

**Fig. 3:**
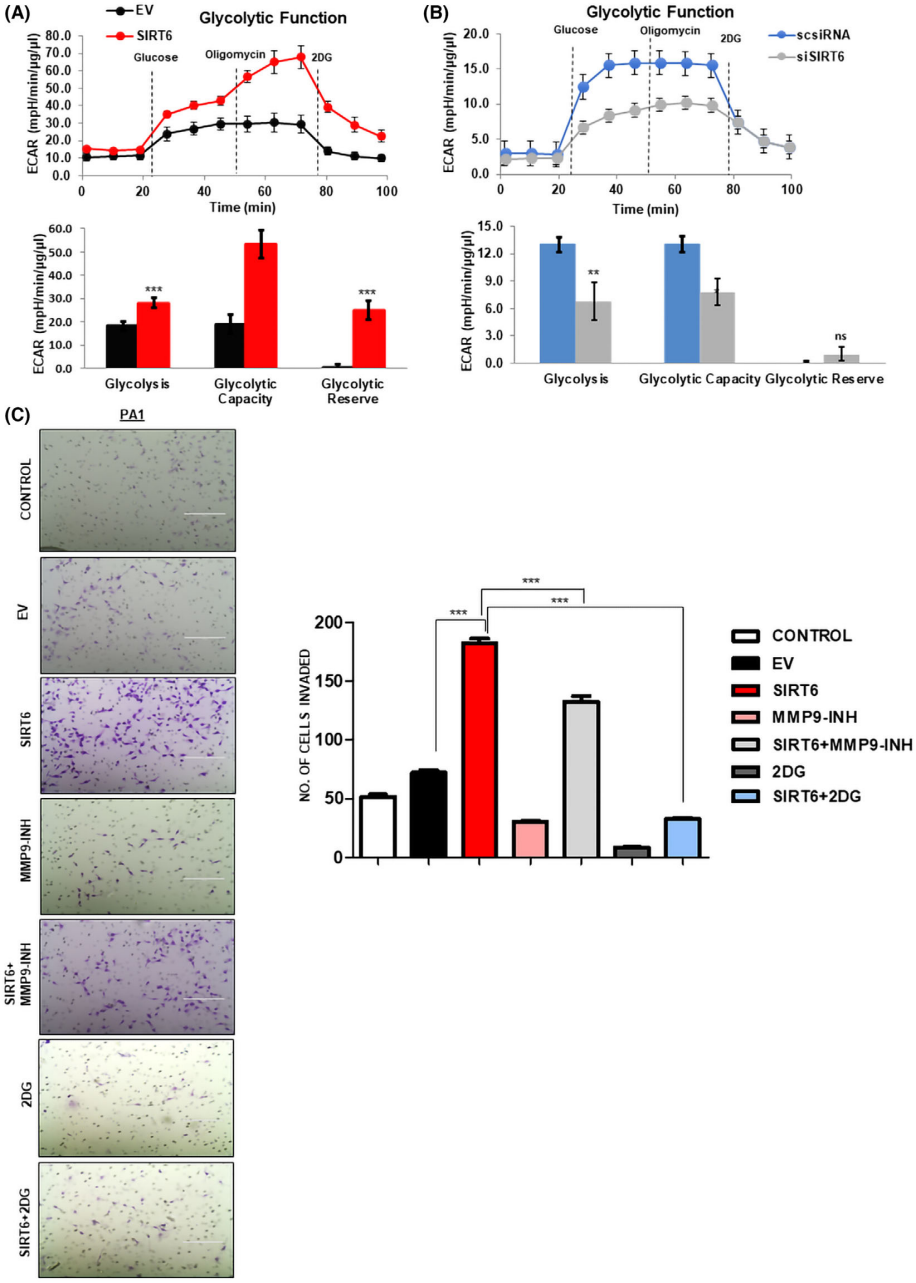
Overexpression of SIRT6 leads to a glycolytic phenotype in ovarian cancer. (A, B) Seahorse Extracellular flux analysis shows elevated ECAR rate in EV‐transfected *vs*. pcDNA 3.1‐SIRT6‐transfected PA1 cells and decreased ECAR rate in scrambled siRNA *vs*. SIRT6‐silenced sample sets in PA1 cells. (C) Cell invasion assay in Control (untransfected), EV‐transfected, SIRT6‐transfected, MMP9 inhibitor‐treated, SIRT6 + MMP9 inhibitor‐treated, 2DG‐treated, and SIRT6 + 2DG‐treated PA1 cells. Number of invaded cells is plotted as bar graphs. *n* = 3, Scale bar = 200 μm. Paired two‐tailed *t*‐test was done to calculate the *P*‐value. Error bars represent standard error of mean (SEM) from three independent experiments. ns—non‐significant *P‐value < 0.05, **P‐value < 0.01, ***P‐value < 0.001. [Colour figure can be viewed at wileyonlinelibrary.com]

Considering the transcriptomic data obtained, we focused on the involvement of SIRT6 in cancer and metabolism. Different authors depicted that inhibiting glycolysis compromises the migration and invasion capacity of the cells [35]. Initially, we compared the metabolic potential of non‐malignant IOSE‐364, with that of cancerous PA1 and SKOV3 cells. Both ovarian cancer cell lines showed a high oxygen consumption rate and high extracellular acidification rate in comparison with IOSE‐364 cell line (Fig. S2A–F). SIRT6 overexpression in IOSE‐364 cells showed increased glycolysis and glycolytic capacity and cells were found to shift toward a more energetic and glycolytic phenotype as compared to EV‐transfected IOSE‐364 cells (Fig. S2G–J). To screen the effect of SIRT6 on glycolysis in ovarian cancer cells, we performed extracellular flux analysis and found that SIRT6 overexpression had significantly increased glycolysis and glycolytic reserve in PA1 cells (Fig. 3A). PA1 cells on SIRT6 overexpression showed an increased OCR/ECAR ratio (Fig. S2K). In contrast, SIRT6 knockdown significantly decreased the extracellular acidification rate (ECAR) in PA1 cells (Fig. 3B). As lower pH in cancer cells is known to facilitate cancer cell invasion, we checked the effect of MMP9 inhibitor and glycolysis inhibitor, 2DG on cancer cell invasiveness [33]. MMP9 inhibitor and glycolytic inhibitor 2‐deoxy‐D‐glucose (2DG) showed decreased cellular invasion which was found to recover when SIRT6 was overexpressed in those sample sets (Fig. 3C). This set of data suggests that SIRT6 regulates the metabolic flux in the cancer cells by increasing metabolic rate which might facilitate cell invasion.

### SIRT6 modulates mitochondrial respiration and morphology

**Fig. 4:**
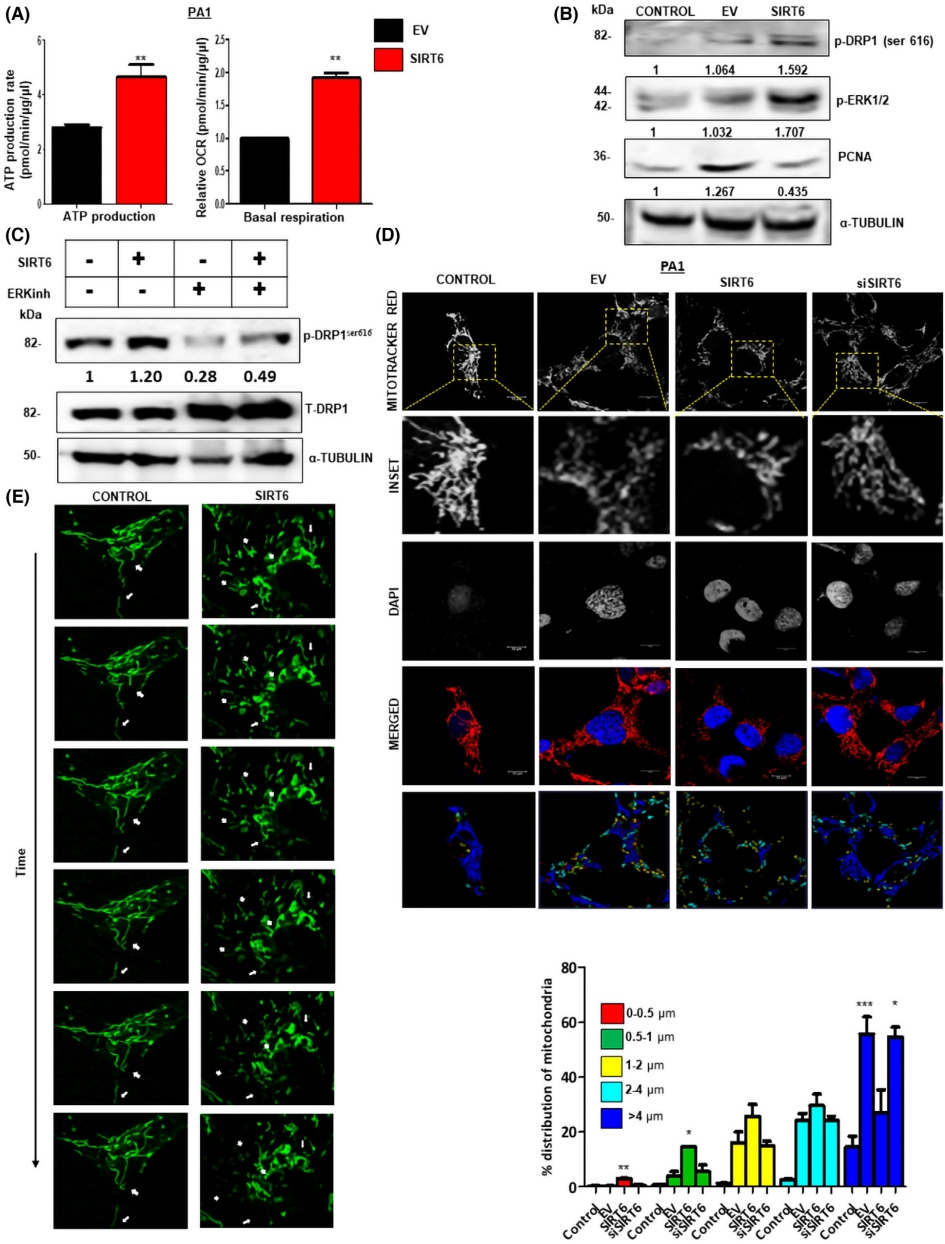
SIRT6 affects mitochondrial morphology through DRP1 phosphorylation. (A) Seahorse extracellular flux analysis depicts higher ATP production and Basal respiration in EV and SIRT6‐transfected PA1 cells. *n* = 3, Paired two‐tailed *t*‐test was done to calculate *P*‐value. (B) Western blot analysis shows p‐DRP1^ser616^, p‐ERK1/2, and PCNA levels in Control (untransfected), EV‐transfected, and pcDNA 3.1‐SIRT6 in PA1 cell line. α‐tubulin was used as gel loading control. (C) Western blot analysis of phospho‐DRP1^ser616^, total DRP1 and α‐tubulin in EV, pcDNA 3.1‐SIRT6, EV + ERK inhibitor, and pcDNA 3.1‐SIRT6 + ERK inhibitor‐treated cells. α‐tubulin was used as gel loading control. (D) Confocal microscopy with Mitotracker red (mitochondria) and DAPI (nuclei) in Control (untransfected), EV, pcDNA 3.1‐SIRT6, and SIRT6‐silenced PA1 cell line. Mitochondrial length distribution over the sample sets was measured using Leica software. (E) Live videography with Mitotracker Green AF488 in Control (untransfected) PA1 and SIRT6‐transfected PA1 cells shown as time‐dependent images. Scale bar = 10 μm. *n* = 3, ANOVA was done to calculate the *P*‐value. Error bars represent standard error of mean (SEM) from three independent experiments. ns—non‐significant, *P‐value < 0.05, **P‐value < 0.01, ***P‐value < 0.001. [Colour figure can be viewed at wileyonlinelibrary.com]

The transcriptomics data analysis suggested that the expression of genes related to mitochondrial function is modulated by SIRT6 overexpression. Mitochondria hold a crucial place in balancing metabolic alterations and tumor cell invasion in cancer cells [5]. On analyzing mitochondrial bioenergetics through the Extracellular flux Analyzer, we found that overexpression of SIRT6 increased the basal respiration and ATP production in PA1 cells (Fig. 4A). Western blot analysis showed increased p‐DRP1^ser616^ (activatory) protein expression on SIRT6 overexpression as compared to control (untransfected) and empty vector (control) cells (Fig. 4B). To speculate on the possible protein kinase that phosphorylates DRP1 at serine‐616, several pathways were taken into consideration. We found no significant change in the expressions of phospho‐p38 MAPK, phospho‐mTOR, phospho‐AMPKα, phospho‐JNKII, and phospho‐CAMKII at 24 h (data not shown). Also, there was no effect of Hif1α inhibition on DRP1 phosphorylation, which suggested that Hif1α is not involved in SIRT6‐mediated mitochondrial fission (Fig. S3A,B). However, we found a significant increase in the phosphorylation of ERK1/2 (p‐p42/44 MAPK) at 24 h in the SIRT6‐overexpressed cells (Fig. 4B). Treatment with ERK inhibitor decreased the phosphorylation status of DRP1 (1.2‐fold *vs*. 0.49‐fold) (Fig. 4C). This indicates that the phosphorylation of DRP1 is due to the activation of the ERK1/2 pathway. To further validate this, confocal microscopic images with Mitotracker Red staining showed an increase in fragmented mitochondria on SIRT6 overexpression and fused mitochondria on SIRT6 knockdown. SIRT6 overexpression increased the number of fragmented smaller mitochondria (~ 2 μm), whereas its knockdown showed the opposite result (Fig. 4D). Confocal microscopy with Mitotracker Red and mitochondrial protein staining was carried out to understand the subcellular localization of these proteins. While p‐DRP1^ser616^ had a significant increase in total fluorescence and mitochondrial colocalization, there was no change in MFN2 fluorescence intensity and mitochondrial colocalization (Fig. S3C–I). The MFN1 fluorescence intensity was reduced without any change in colocalization statistics with mitochondria (Fig. S3C–I). qPCR analysis showed decreased expression of OPA1 and an increase in FIS1 mRNA levels in PA1 cells (Fig. S3J). These results suggest that SIRT6 overexpression is responsible for inducing mitochondrial fragmentation through DRP1 activation at serine‐616.

Further, mitochondrial dynamics were studied through live confocal microscopy with Mitotracker Green staining. Videography depicted mitochondrial fragmentation in SIRT6‐overexpressed PA1 cells (Video S2), whereas control PA1 cells (Video S1) showed unchanged mitochondrial dynamics, in a time‐dependent manner (Fig. 4E).

### SIRT6‐induced changes in mitochondrial morphology cause its localization along actin filaments

**Fig. 5:**
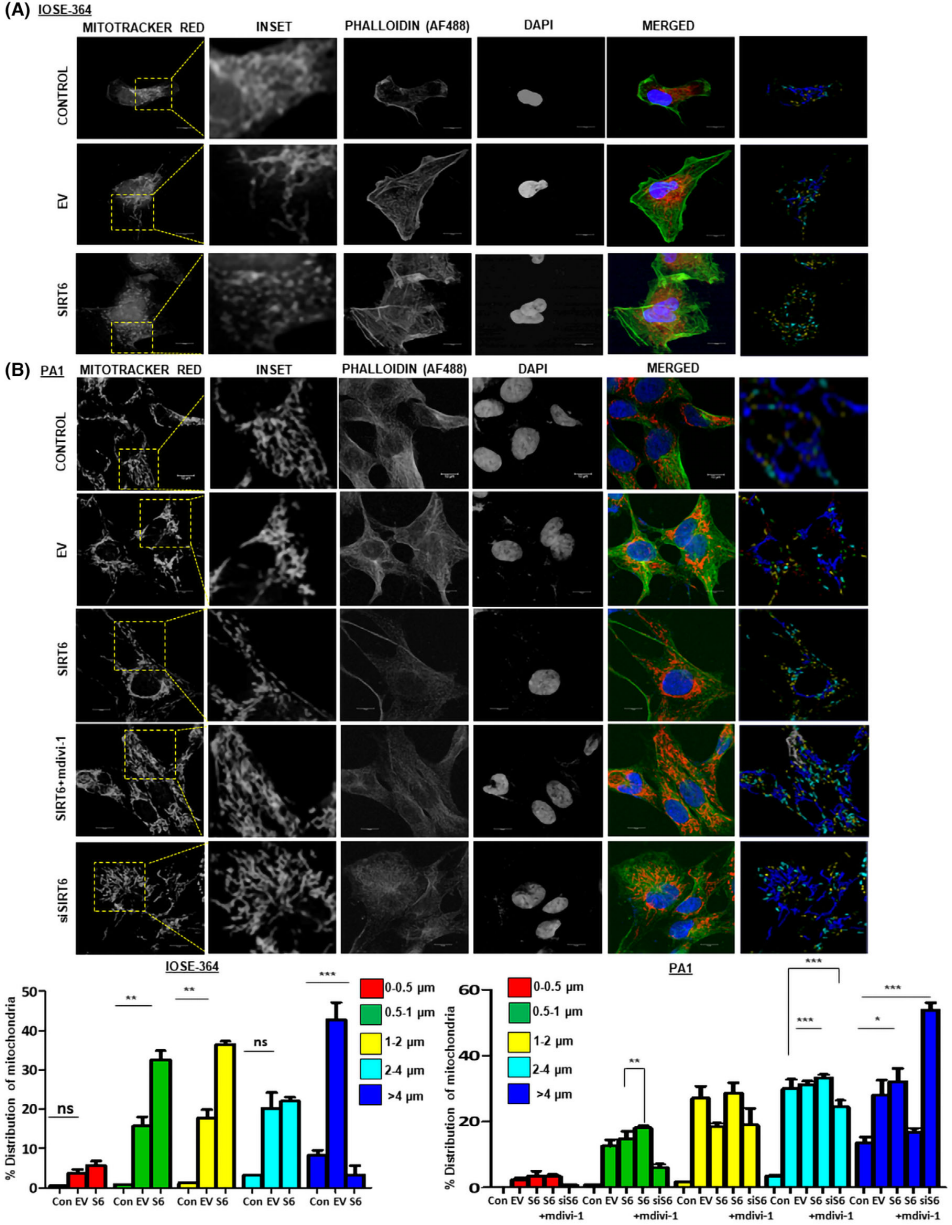
SIRT6 directs mitochondrial fission by augmenting actin polymerization. (A, B) Confocal microscopy in Control (untransfected), EV and pcDNA 3.1‐SIRT6‐transfected IOSE‐364 and PA1 cells with Phalloidin Green AF488 (actin), Mitotracker Red (mitochondria), and DAPI (nuclei). Bar graphs depicted for calculating mitochondrial length using Leica software. Scale bar = 10 μm. *n* = 3, ANOVA was done to calculate the *P*‐value. Error bars represent standard error of mean (SEM) from three independent experiments. ns—non‐significant *P‐value < 0.05, **P‐value < 0.01, ***P‐value < 0.001. [Colour figure can be viewed at wileyonlinelibrary.com]

The increase in mitochondrial fission mediated by DRP1 contributes to cancer cell invasion. Cancer cell invasion and movement involve a coordinated rearrangement of cancer cells with the cytoskeleton to form invadopodia‐like structures [50]. A combination of AF488‐tagged‐Phalloidin and Mitotracker Red stain revealed increased actin stress fiber formation aligning together with fragmented mitochondria in both IOSE‐364 and PA1 cells overexpressed with SIRT6 (Fig. 5A,B). siRNA‐mediated SIRT6 knockdown and mdivi‐1 treatment reduced the mitochondrial fragmentation and actin stress fiber formation in PA1 cells (Fig. 5B).

### SIRT6‐mediated mitochondrial alterations lead to oncogenesis

**Fig. 6:**
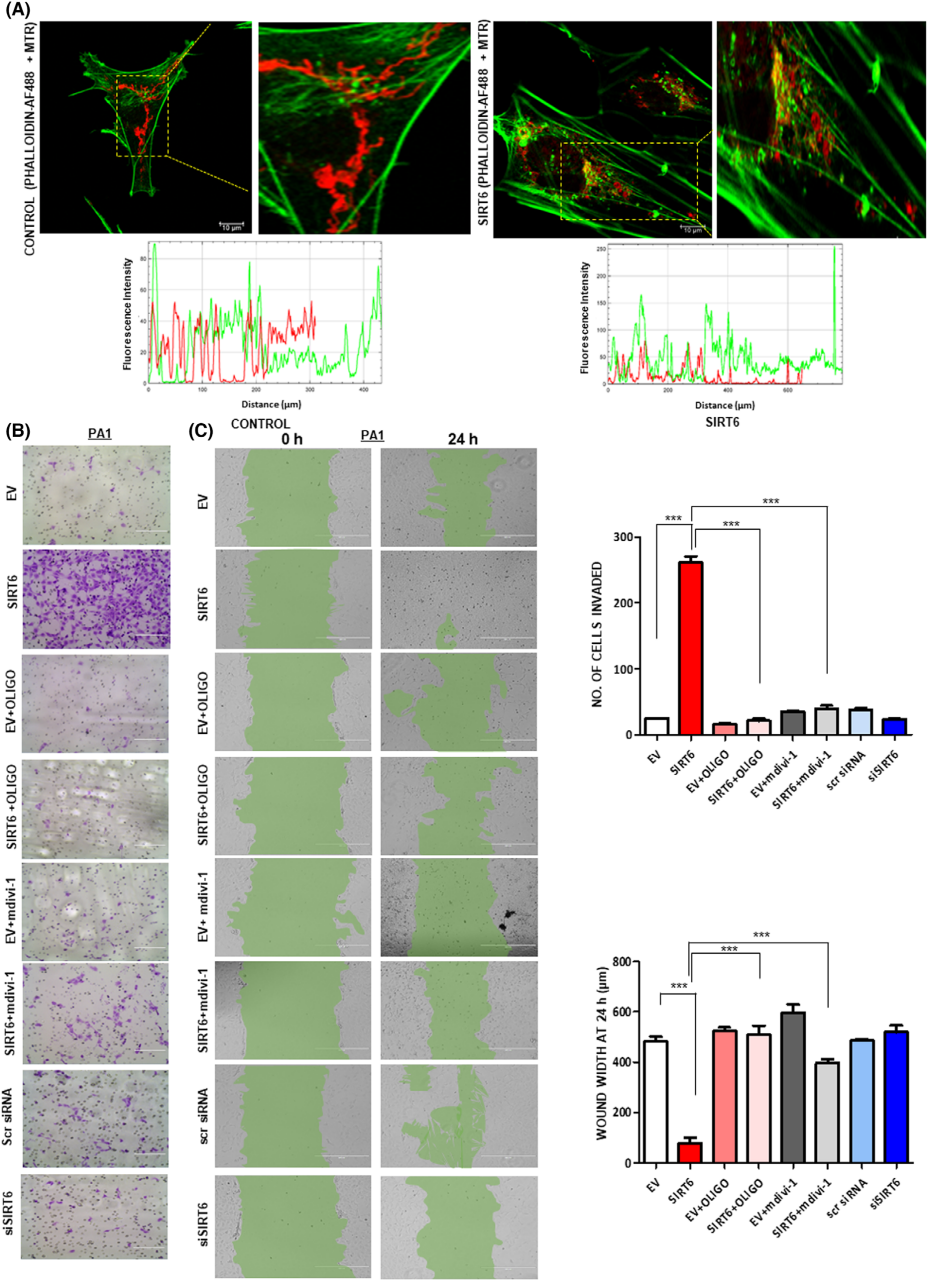
SIRT6 enhances ovarian cancer cell migration and invasion through mitochondrial fission. (A) Phalloidin Green AF488 + Mitotracker Red staining in Control (untransfected) and SIRT6‐transfected PA1 cells was carried out. Scale bar = 10 μm, Line‐scan analysis between Phalloidin Green AF488 and Mitotracker Red stained images was done using ImageJ software. (B) Matrigel invasion studies to show the number of invaded cells in sample sets of EV, pcDNA 3.1‐SIRT6, EV+ Oligomycin‐A, pcDNA 3.1‐SIRT6 + Oligomycin‐A, EV + mdivi‐1, pcDNA 3.1‐SIRT6 + mdivi‐1, scrambled siRNA, and SIRT6 siRNA. Scale bar = 200 μm. (C) Wound healing assay to show the distance after wound healing in sample sets EV, pcDNA 3.1‐SIRT6, EV+ Oligomycin‐A, pcDNA 3.1‐SIRT6 + Oligomycin‐A, EV + mdivi‐1, pcDNA 3.1‐SIRT6 + mdivi‐1, scrambled siRNA, and SIRT6 siRNA. Scale bar = 400 μm. *n* = 3, ANOVA was done to calculate the *P*‐value. Error bars represent standard error of mean (SEM) from three independent experiments. ns—non‐significant *P‐value < 0.05, **P‐value < 0.01, ***P‐value < 0.001. [Colour figure can be viewed at wileyonlinelibrary.com]

Mitochondrial fission is associated with increased metastatic potential in cancer cells [50]. To elaborate on this characteristic feature, a more clarified visualization of thicker, longer, and highly oriented F‐actin filament bundles that converge with fragmented mitochondria was observed in SIRT6‐overexpressed cells. The line‐scan analysis further confirms this observation of increased fluorescence intensity as a measure of more prominent F‐actin visualization and the presence of longer filaments. Also, fragmented mitochondria were observed to be distributed through longer distances along with actin filaments distribution under SIRT6‐overexpressed conditions (Fig. 6A). We have found that SIRT6 overexpression increases the number of invaded cells by Matrigel invasion assay and cell migration through wound healing assay while SIRT6 knockdown decreased the same (Fig. 6B,C). Inhibition of mitochondrial fragmentation with mdivi‐1 and mitochondrial ATP production with oligomycin‐A decreased the migratory and invasive properties manifested by SIRT6 overexpression (Fig. 6B,C). Thus, these data suggest that SIRT6 induces mitochondrial fragmentation and helps in facilitating directional actin formation important for cancer cell invasion.

## Discussion

Among gynecological cancers, ovarian cancer is asymptomatic as it is chiefly diagnosed at later stages when it has already metastasized to vital organs. Research has been carried out centering on different molecular players in this regard to understand the possible mechanism. In this report, we have focused on a crucial enzyme of the sirtuin family SIRT6, whose role in cancer progression has been conflicting in different cancer types [19]. SIRT6 is involved in several areas of oncogenesis such as glucose metabolism, genomic stability and DNA repair, cell survival and inflammation [51]. The lack of direct evidence regarding the exact role of SIRT6 in ovarian cancer progression led us to decipher its association in driving ovarian cancer cell invasion.

Cell proliferation and metastases are both major contributing factors that support tumor cell growth. Through qPCR experiments and histology studies, we configured the increased level of SIRT6 in ovarian cancer cells (PA1) as well as in patients' tissues. Ovarian cancer cells (PA1) overexpressing SIRT6 were found to show unaltered proliferation depicted by the unchanged expression of the cell proliferation markers PCNA and Ki‐67. However, there was a marked elevation in cell invasion properties in both PA1 and SKOV3 ovarian cancer cells on SIRT6 overexpression, which was found to decrease subsequently on SIRT6 knockdown. Invading cancer cells need to constantly reprogram their metabolism in response to fluctuating energy requirements in the tumor microenvironment. Reduced or unaltered cell proliferation is essential for cancer cells that have undergone EMT programs, as enhanced proliferation is antagonistic to cytoskeletal rearrangement that is characteristic of cell invasion and movement. Disseminating tumor cells manifest lower proliferation rates and resistance to apoptosis to sustain in hostile environments before successful secondary site establishment [52, 53]. Increased expression of matrix metalloproteases (MMPs) is a characteristic of cancer cells undergoing invasion, and in this study, we found higher levels of MMP9 that have been documented to be regulated by higher SIRT6 status in cancer cells [20, 54]. Transcriptomics data revealed the effect of SIRT6 on majorly three groups *viz*., cancer, metabolism, and mitochondria, which gave us an indication as to how it may modulate ovarian cancer. Other than this information, gene ontology suggests the involvement of SIRT6 in protein transport, transcriptional regulation, protein binding, and various signal transduction processes.

To sustain survival advantage, cancer cells often need to rewire their metabolic phenotype. They rely on rapid cellular glycolysis and oxidative phosphorylation to meet their energetic demands. In general, ovarian cancer cell lines PA1 and SKOV3 both depict high ECAR and OCR when compared to the non‐malignant IOSE‐364 cell line. Accordingly, the sirtuin group of enzymes also has metabolic repercussions that further intensify tumor progression [21]. SIRT6 overexpression exhibited augmented glycolytic rate whereas silencing SIRT6 decreased glycolysis in ovarian cancer cells. Enhanced glycolysis leads to the accumulation of lactate, an “oncometabolite,” inside tumor cells. This lactate is further secreted to the extracellular medium, which acidifies the tumor microenvironment causing a decrease in pH to around 6–6.5 [32]. This acidification drives the increased expression and activation of matrix metalloproteases (MMP2 and MMP9), which proteolytically cleaves extracellular matrix proteins driving matrix degradation and tumor cell invasion [41]. A shift in oxygen consumption rate (OCR) is inevitable under such circumstances to support the changing ECAR rate. Accordingly, we found an enhanced rate of basal respiration by SIRT6 in ovarian cancer cells. This metabolic plasticity is associated with the ever‐changing dynamic mitochondria, which are the seat of oxygen consumption.

Mitochondria are distributed throughout the cell stretching from the nuclear periphery to the plasma membrane [55]. The localization of mitochondria varies under different conditions depending on ATP requirement, nutrient deprivation, starvation, ROS accumulation, *etc*. Mitochondrial dynamics include fission and fusion that helps to maintain mitochondrial distribution, size and shape [13]. In tumor cells, mitochondria play a crucial role in facilitating tumor cell migration and invasion. Cell migration is an energy‐consuming process that entails cellular reshaping and translocation. Tumor cells undergoing migration form lamellipodia with mitochondrial accumulation at the leading edge of moving cells. The actin cytoskeletal rearrangement that takes place during cell invasion is bio‐energetically challenging due to the higher requirement of ATP near the vicinity. Moving cells have directional actin fibers alongside fragmented mitochondria at their focal points [12]. Actively respiring mitochondria translocate to the lamellipodia periphery and fuel actin polymerization in these locations. In contrast, mitochondria with compromised oxidative phosphorylation cannot relocate to the cellular periphery and thereby cells lose their migratory and invasive property [36, 56]. The formation of longer and directional actin fiber formation is also facilitated by lower pH of 6–6.5, which arises in cells undergoing higher glycolysis [57]. The translocation of mitochondria in the cells occurs via the Miro‐Milton transport complex that directs the kinesin‐dependent movement of mitochondria. Miro is a linker rho‐GTPase‐1 (miro‐1) between mitochondria and microtubules that assists in orienting mitochondria toward the plasma membrane [58]. This axis is fueled by the presence of glucose and calcium flux in the vicinity. Reports also suggest that the localization of functional mitochondria is influenced by glucose levels in the tumor cells. In faster migratory cells, glucose gets accumulated near the peripheral regions accompanied by mitochondrial enrichment at those sites [59].

Another crucial factor for mitochondrial localization in cells is attributed to reactive oxygen species (ROS) production. Mitochondria actively generating ROS relocate near the nucleus and stimulate the expression of genes with HRE in their promoter regions. Inhibition of perinuclear clustering of mitochondria was not found to affect Hif1α accumulation in the nucleus under such conditions [60]. Furthermore, we have previously reported that upon glutamine starvation in PA1 cells, mitochondrial fragmentation is promoted. These fragmented mitochondria relocate perinuclearly, increasing ROS levels in the nucleus [39]. In contrast in this report, we have found an increase in Hif1α mRNA levels without any change in the protein level. There was a slight decrease in ROS production levels on SIRT6 overexpression (not shown) in PA1 cells, and this further justifies our finding that mitochondria localize near the actin foci. The elevated mitochondrial number is facilitated by mitochondrial fragmentation, characteristic mitochondrial fission mediated by dynamin‐related protein 1 (DRP1) [56, 61]. SIRT6 modulates activatory phosphorylation of DRP1^ser616^ which brings about both increased mitochondrial fragmentation and localization. The presence of higher p‐DRP1^ser616^ in the mitochondrial surface indicates increased functionality of this protein in promoting fragmentation. Knockdown of mitochondrial fission genes like *DRP1* decreases both the velocity of mitochondrial movement and its directionality toward the anterior edges of cancer cells [10, 36]. Reports indicate that extracellular signal regulatory kinase 1/2 (ERK1/2) regulates the levels MMPs and inhibits intrinsic apoptosis pathways in hepatic cancer, non‐small cell lung carcinoma, and colorectal cancer [30]. Mechanistically, we found extracellular signal regulatory kinase 1/2 (ERK1/2) to phosphorylate DRP1 protein at serine‐616 causing its activation. ERK1/2 is a well‐known “Drp kinase” and is known to stimulate phosphorylation in response to different growth factors, transcription factors, and enzymes [30]. Beyond that, the MAPK signaling pathway also modulates glycolysis and this could be the reason for the increased cellular glycolysis on SIRT6 overexpression in ovarian cancer cell lines [31]. Inhibition of ERK phosphorylation abrogates DRP1 phosphorylation at serine‐616, and this further indicates the involvement of ERK in DRP1 activation [39].

In this report, we have indicated for the first time, the involvement of SIRT6 in illustrating the importance of mitochondria to facilitate tumor progression through altered actin dynamics. To establish this connection, we treated cells with two widely used inhibitors, oligomycin‐A (mitochondrial complex V inhibitor) and mdivi‐1 (mitochondrial fission inhibitor) and found that when mitochondrial function is compromised, both invasion and migration of these cancer cells are abrogated [61, 62, 63]. Phalloidin‐Mitotracker Red staining further corroborated our findings of localized actin foci in SIRT6‐transfected cells. However, a disoriented mitochondria‐actin association was observed in mitochondrial functional and structural inhibitor treatment as well as SIRT6‐knockdown cells. This polarized trafficking of mitochondria is an important characteristic of motile cancer cells.

## Conclusion

SIRT6 is a predominant member of the sirtuin family having implications for tumor metabolism and progression. It increases glycolysis and mitochondrial respiration making the cancer cells privileged with ATP. This ATP further facilitates tumor cell migration and invasion while mitochondrial inhibitors revoke this cellular motion. Overall, our study puts forward the tumor‐promoting function of SIRT6 in ovarian cancer cell lines and high‐grade patient samples. We have evidenced that fragmentation of mitochondria accompanied by changes in ECAR facilitate ovarian cancer cell invasion. On silencing SIRT6 with its siRNA, there was a decrease in mitochondrial fragmentation along with reduced cancer cell migration and invasion. Further, *in vivo* studies might pave the way to substantiate our experimental data such that therapeutic approaches with probable inhibitors can be designed in future.

## Conflict of interest

The authors declare no conflict of interest.

## Author contributions

SSR, SB, and PP conceptualized this study and reviewed and drafted the manuscript. SB and PP designed and performed experiments and analyzed the data. UR performed experiments and reviewed the manuscript. DDG provided patient tissue slides and performed experiments.

## Supporting information


**Fig. S1.** Aberrant SIRT6 expression in ovarian cancer contributes to tumor invasion (A) Haematoxylin‐Eosin staining of normal ovary and high‐grade carcinoma patient tissue sections. Scale bar=20 μm. (B) Kaplan‐Meier survival plot w.r.t SIRT6 was obtained for 373 patient samples from TCGA data sets. (C) Western blot analysis showing SIRT6 protein level in PA1 after SIRT6 overexpression vs. EV(control). Relative protein expression is depicted in bar graphs, n=3. SIRT6 has been normalized to α‐tubulin levels. (D) qPCR data showing significant decrease in SIRT6 expression on scrambled siRNA‐transfected vs. SIRT6‐siRNA transfected PA1 cells, n=3. (E) Matrigel invasion studies show higher number of invaded cells in SIRT6‐overexpressed cells w.r.t control, EV‐transfected and SIRT6‐silenced sample sets in SKOV3 cells. n=3, Scale bar=100 μm. One‐way ANOVA was done to calculate the P value. Error bars represent standard error of mean (SEM) from three independent experiments. * (P‐value<0.05), ** (P‐value<0.01), *** (P‐value<0.001).
**Fig. S2.** (A) OCR/ECAR phenotype of IOSE‐364 vs. PA1 cell line, n=2. (B, C) Respective Basal OCR and ECAR in IOSE‐364 and PA1 cell line. (D) OCR/ECAR phenotype of IOSE‐364 vs. SKOV3 cell line (E, F) Respective Basal OCR and ECAR in IOSE‐364 vs. SKOV3 cell line, n=2. (G,H) Glycolysis and Glycolytic capacity of EV‐transfected and SIRT6‐transfected IOSE‐364 cells, n=3. (I) Energy phenotype of EV vs. SIRT6‐transfected IOSE‐364 cell line. (J) Ratio of OCR/ECAR in EV vs. SIRT6‐transfected IOSE‐364 cell line. (K) Ratio of OCR/ECAR in EV vs. SIRT6‐transfected PA1 cell line. n=3, Paired two‐tailed t‐test was done to calculate the P value. Error bars represent standard error of mean (SEM) from three independent experiments. * (P‐value<0.05), ** (P‐ value<0.01), ***(P‐value<0.001).
**Fig. S3.** (A) qPCR analysis showed increased HIF1α mRNA levels in EV‐transfected and SIRT6‐transfected PA1 cells (n=3). (B) p‐DRP1ser616 expression w.r.t total‐DRP1 was checked in the presence of HIF1α‐siRNA through western blot studies. (C) Confocal microscopy staining with Mitotracker Red and p‐DRP1ser616‐AF488 in EV, SIRT6 and SIRT6‐siRNA transfected PA1 cells. n=3, Scale bar=10 μm. (D) Calculation of colocalization between Mitotracker Red and p‐DRP1ser616‐AF488 in EV, SIRT6 and SIRT6‐siRNA transfected PA1 cells. Mean fluorescent intensity (fold change) of p‐DRP1ser616‐AF488. (E) Confocal microscopy staining with Mitotracker Red and MFN1‐AF488 in Control (untransfected), EV, SIRT6 and SIRT6‐siRNA Transfected PA1 cells. n=3, Scale bar=10 μm. (F) Calculation of colocalization between Mitotracker Red and MFN1‐AF488 in EV, SIRT6 and SIRT6‐siRNA transfected PA1 cells. (G) Confocal microscopy staining with Mitotracker Red and MFN2‐AF488 in Control (untransfected), EV, SIRT6 and SIRT6‐siRNA transfected PA1 cells. n=3, Scale bar=10 μm. (H) Calculation of colocalization between Mitotracker Red and MFN2‐AF488 in EV, SIRT6 and SIRT6‐siRNA transfected PA1 cells. (I) Mean Fluorescent Intensity (fold change) between Control (untransfected), EV‐transfected and SIRT6‐transfected for MFN1‐AF488 and MFN2‐AF488 SIRT6‐transfected PA1 cells (n=3). (J) qPCR analysis to check mRNA levels of FIS1 and OPA1 in EV vs. SIRT6‐transfected PA1 cells. n=3, Paired two‐tailed t‐test was done to calculate the P‐value. Error bars represent standard error of mean (SEM) from three independent experiments. * (P‐value<0.05), ** (P‐value<0.01), *** (P‐value<0.001).
**Fig. S4.** (A,B) Full blot of Figure 1D. (C) Full blot of Supplementary Figure 1C.
**Fig. S5.** (A–D) Full blots of figure 4B. (E–G) Full blot of Figure 4C.
**Fig. S6.** (A,B) Full blot of Supplementary Figure 3B.

**Video S1.** Live Videography of Control PA1 cells stained with Mitotracker Green.

**Video S2.** Live Videography of SIRT6‐overexpressed PA1 cells stained with Mitotracker Green.

## Data Availability

The data that support the findings of this study are available from the corresponding author (sibsankar@iicb.res.in) on reasonable request. The transcriptomics data have been deposited in the NCBI SRA portal with BioProject ID PRJNA783744 and are openly available with this URL (https://www.ncbi.nlm.nih.gov/bioproject/?term=PRJNA783744).
