# Supplementary material for: SIRT6 promotes mitochondrial fission and subsequent cellular invasion in ovarian cancer
Source: FEBS Open Bio. 2022 Jun 24;12(9):1657–76. doi: 10.1002/2211-5463.13452 (PMC9433826; doi:10.1002/2211-5463.13452)
Supplement: Supplementary file 1 — Fig. S1. Aberrant SIRT6 expression in ovarian cancer contributes to tumor invasion (A) Haematoxylin‐Eosin staining of normal ovary and high‐grade carcinoma patient tissue sections. Scale bar=20 μm. (B) Kaplan‐Meier survival plot w.r.t SIRT6 was obtained for 373 patient samples from TCGA data sets. (C) Western blot analysis showing SIRT6 protein level in PA1 after SIRT6 overexpression vs. EV(control). Relative protein expression is depicted in bar graphs, n=3. SIRT6 has been normalized to α‐tubulin levels. (D) qPCR data showing significant decrease in SIRT6 expression on scrambled siRNA‐transfected vs. SIRT6‐siRNA transfected PA1 cells, n=3. (E) Matrigel invasion studies show higher number of invaded cells in SIRT6‐overexpressed cells w.r.t control, EV‐transfected and SIRT6‐silenced sample sets in SKOV3 cells. n=3, Scale bar=100 μm. One‐way ANOVA was done to calculate the P value. Error bars represent standard error of mean (SEM) from three independent experiments. * (P‐value<0.05), ** (P‐value<0.01), *** (P‐value<0.001). Fig. S2. (A) OCR/ECAR phenotype of IOSE‐364 vs. PA1 cell line, n=2. (B, C) Respective Basal OCR and ECAR in IOSE‐364 and PA1 cell line. (D) OCR/ECAR phenotype of IOSE‐364 vs. SKOV3 cell line (E, F) Respective Basal OCR and ECAR in IOSE‐364 vs. SKOV3 cell line, n=2. (G,H) Glycolysis and Glycolytic capacity of EV‐transfected and SIRT6‐transfected IOSE‐364 cells, n=3. (I) Energy phenotype of EV vs. SIRT6‐transfected IOSE‐364 cell line. (J) Ratio of OCR/ECAR in EV vs. SIRT6‐transfected IOSE‐364 cell line. (K) Ratio of OCR/ECAR in EV vs. SIRT6‐transfected PA1 cell line. n=3, Paired two‐tailed t‐test was done to calculate the P value. Error bars represent standard error of mean (SEM) from three independent experiments. * (P‐value<0.05), ** (P‐ value<0.01), ***(P‐value<0.001). Fig. S3. (A) qPCR analysis showed increased HIF1α mRNA levels in EV‐transfected and SIRT6‐transfected PA1 cells (n=3). (B) p‐DRP1ser616 expression w.r.t total‐DRP1 was checked in the [file FEB4-12-1657-s001.pdf]

SUPPLEMENTARY FIGURE 1

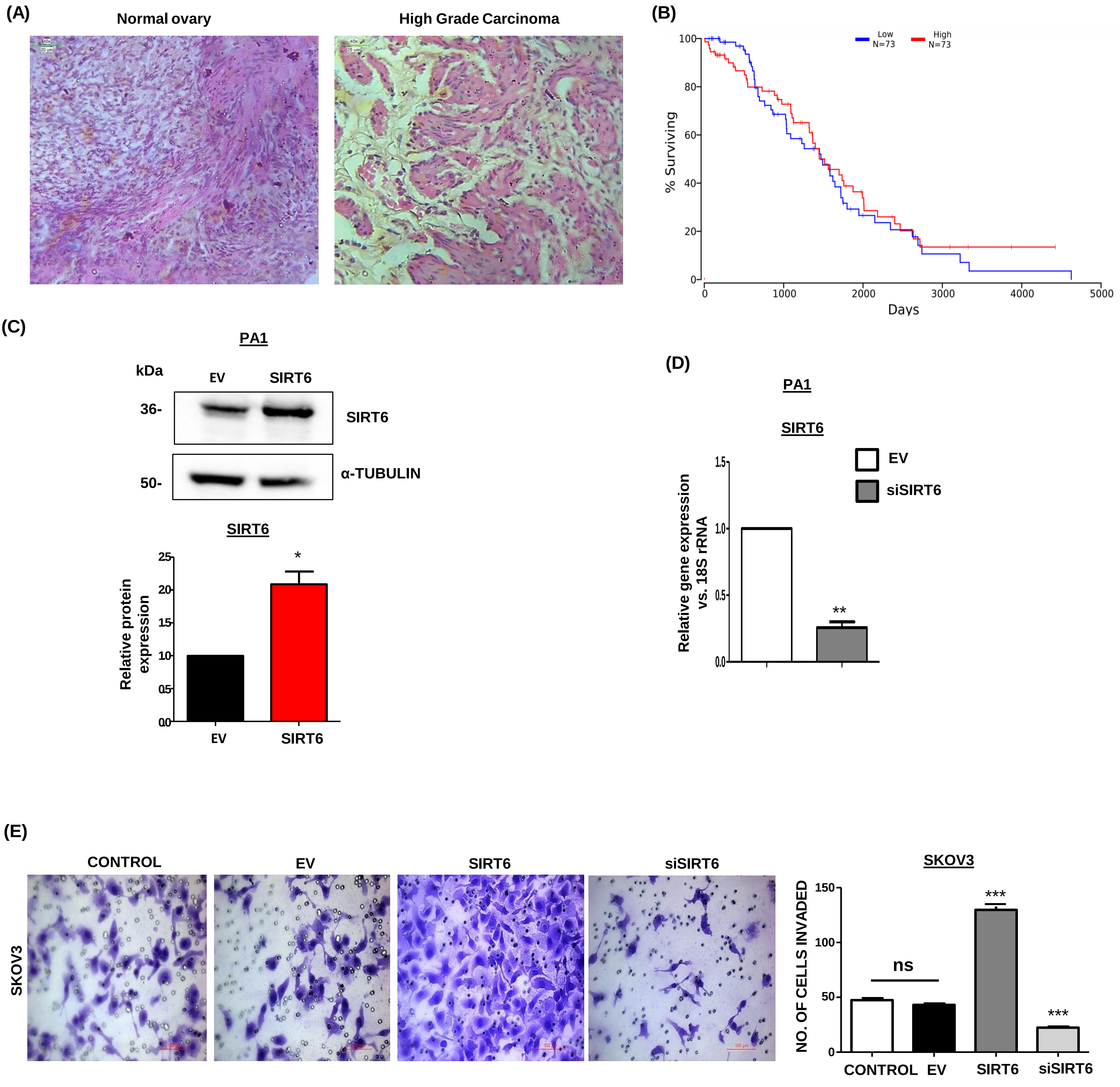

Supplementary Figure 1: Aberrant SIRT6 expression in ovarian cancer contributes to tumor invasion (A) Haematoxylin-Eosin staining of normal ovary and high-grade carcinoma patient tissue sections. Scale bar=20 µm. (B) Kaplan-Meier survival plot w.r.t SIRT6 was obtained for 373 patient samples from TCGA data sets. (C) Western blot analysis showing SIRT6 protein level in PA1 after SIRT6 overexpression vs. EV(control). Relative protein expression is depicted in bar graphs, n=3. SIRT6 has been normalized to α-tubulin levels. (D) qPCR data showing significant decrease in SIRT6 expression on scrambled siRNA-transfected vs. SIRT6-siRNA transfected PA1 cells, n=3. (E) Matrigel invasion studies show higher number of invaded cells in SIRT6-overexpressed cells w.r.t control, EV-transfected and SIRT6-silenced sample sets in SKOV3 cells. n=3, Scale bar=100 µm. One-way ANOVA was done to calculate the P value. Error bars represent standard error of mean (SEM) from three independent experiments. * (P-value<0.05), ** (P-value<0.01), *** (P-value<0.001).

SUPPLEMENTARY FIGURE 2

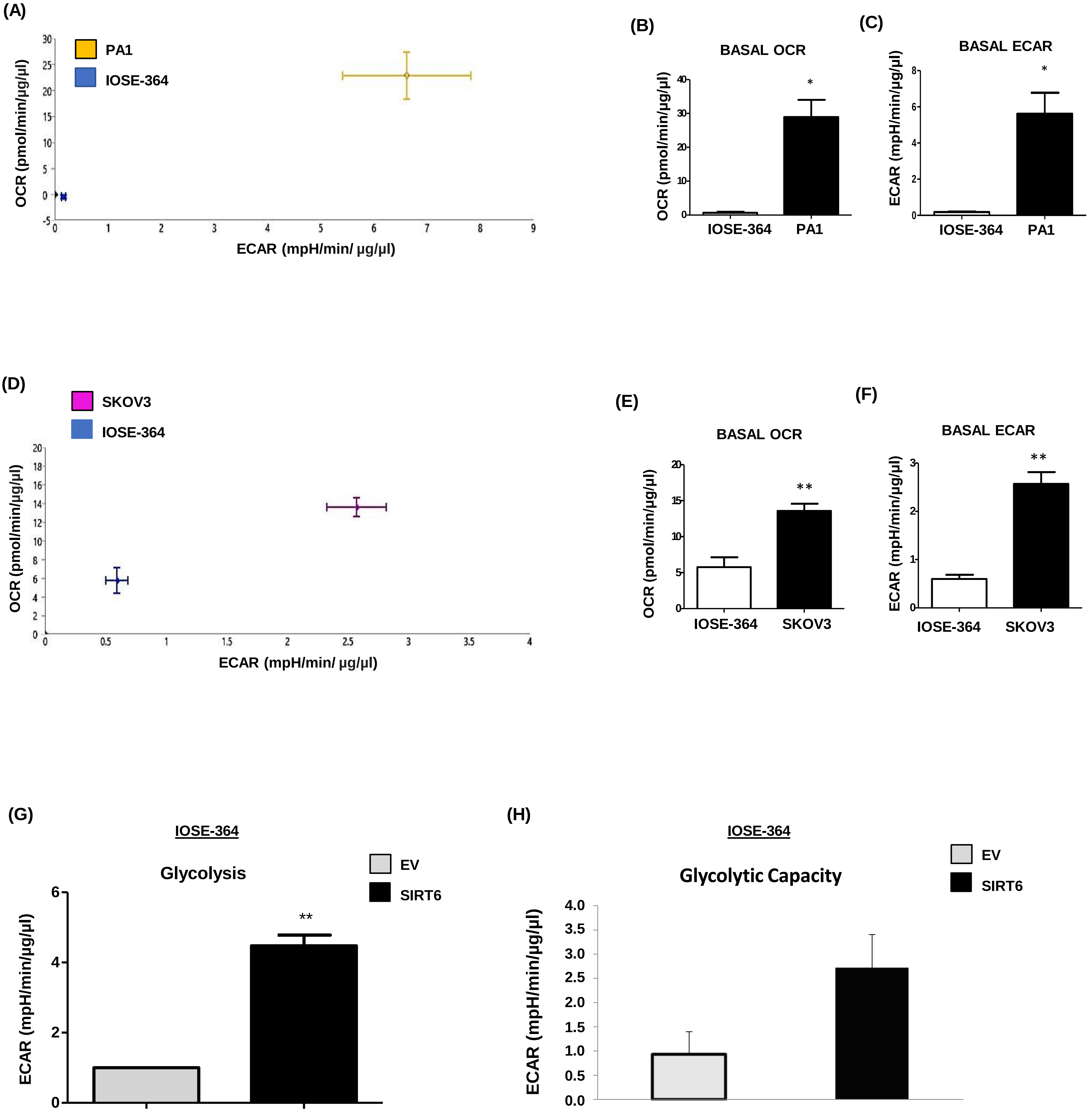

(I)

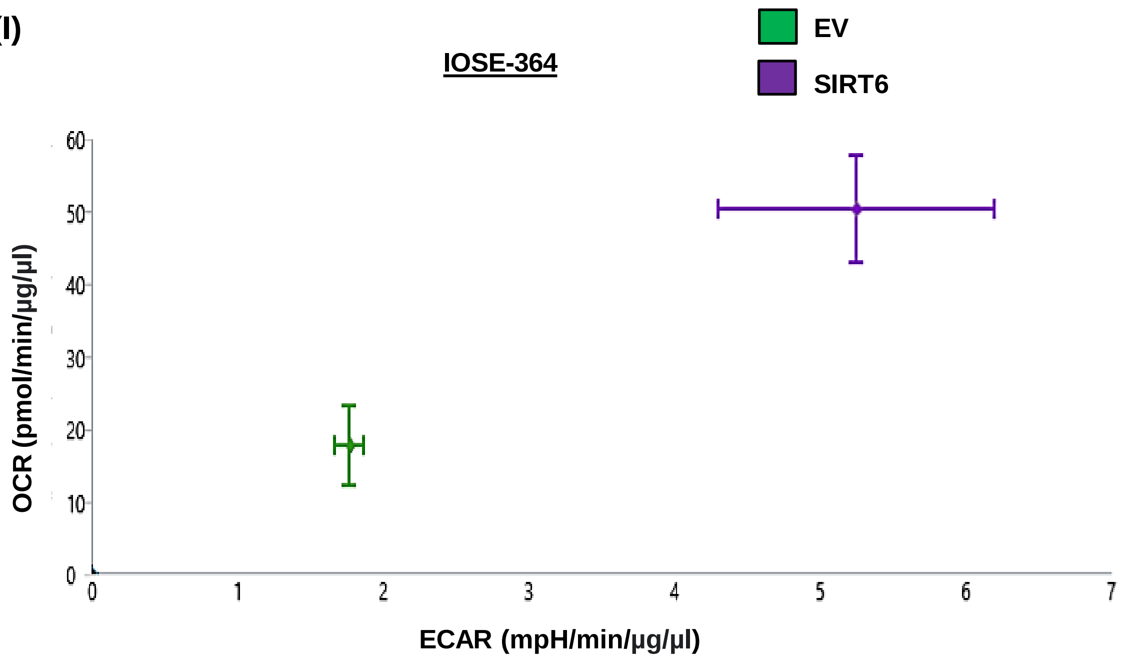

(J)

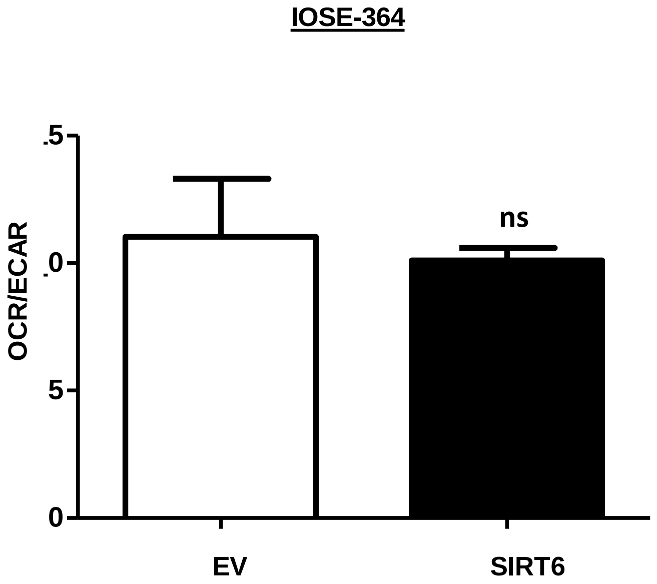

(K)

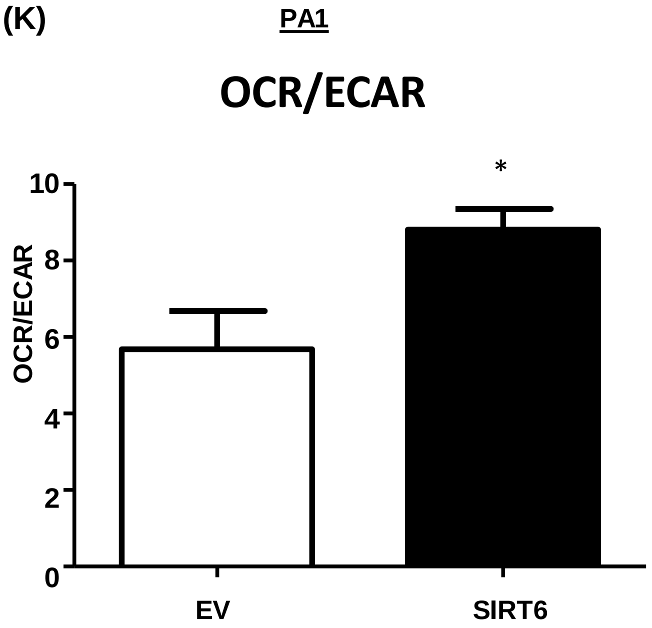

Supplementary Figure 2: (A) OCR/ECAR phenotype of IOSE-364 vs. PA1 cell line, n=2. (B, C) Respective Basal OCR and ECAR in IOSE-364 and PA1 cell line. (D) OCR/ECAR phenotype of IOSE-364 vs. SKOV3 cell line (E, F) Respective Basal OCR and ECAR in IOSE-364 vs. SKOV3 cell line, n=2. (G,H) Glycolysis and Glycolytic capacity of EV-transfected and SIRT6-transfected IOSE-364 cells, n=3. (I) Energy phenotype of EV vs. SIRT6-transfected IOSE-364 cell line. (J) Ratio of OCR/ECAR in EV vs. SIRT6-transfected IOSE-364 cell line. (K) Ratio of OCR/ECAR in EV vs. SIRT6-transfected PA1 cell line. n=3, Paired two-tailed t-test was done to calculate the P value. Error bars represent standard error of mean (SEM) from three independent experiments. * (P-value<0.05), ** (P- value<0.01), ***(P-value<0.001).

SUPPLEMENTARY FIGURE 3

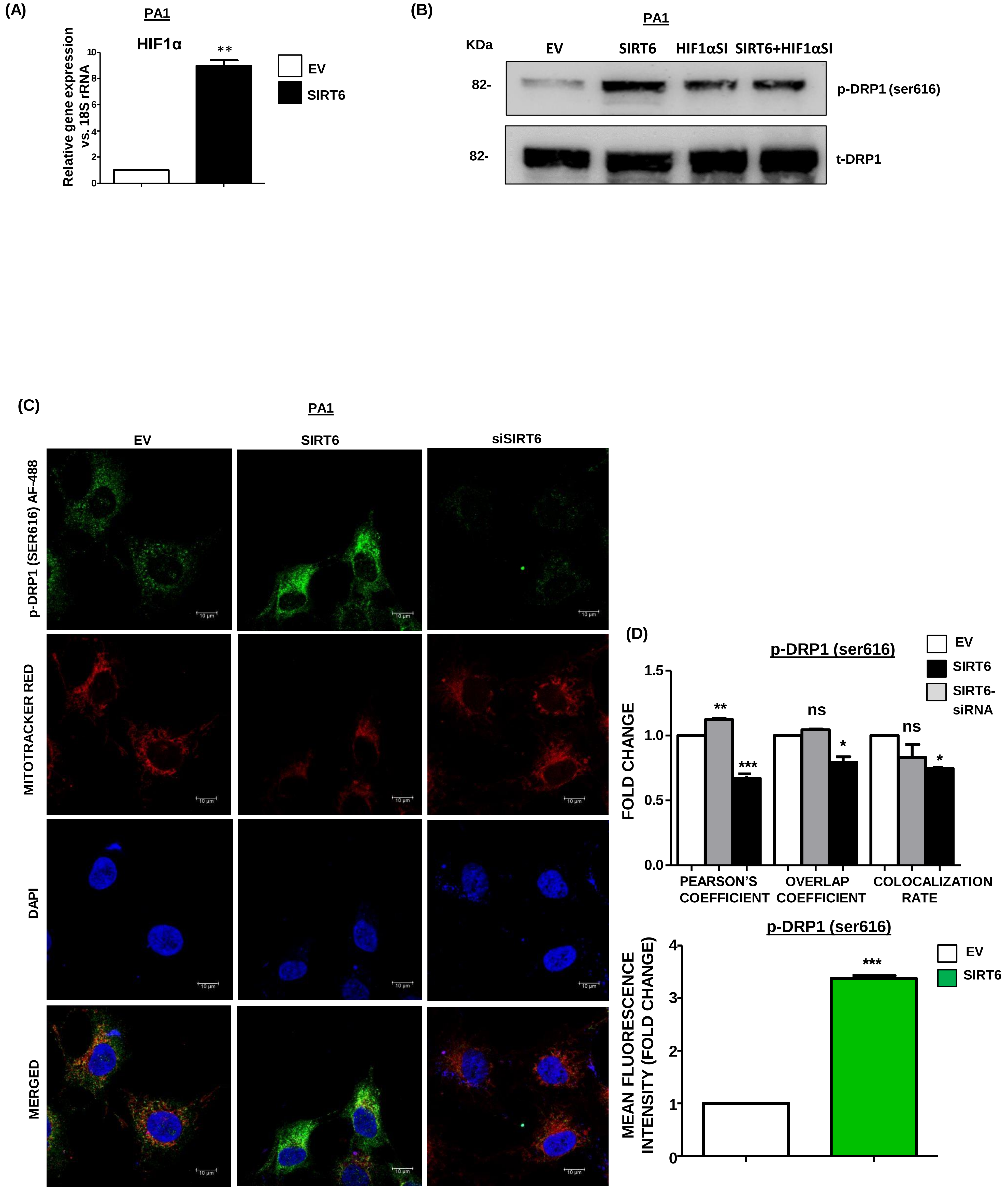

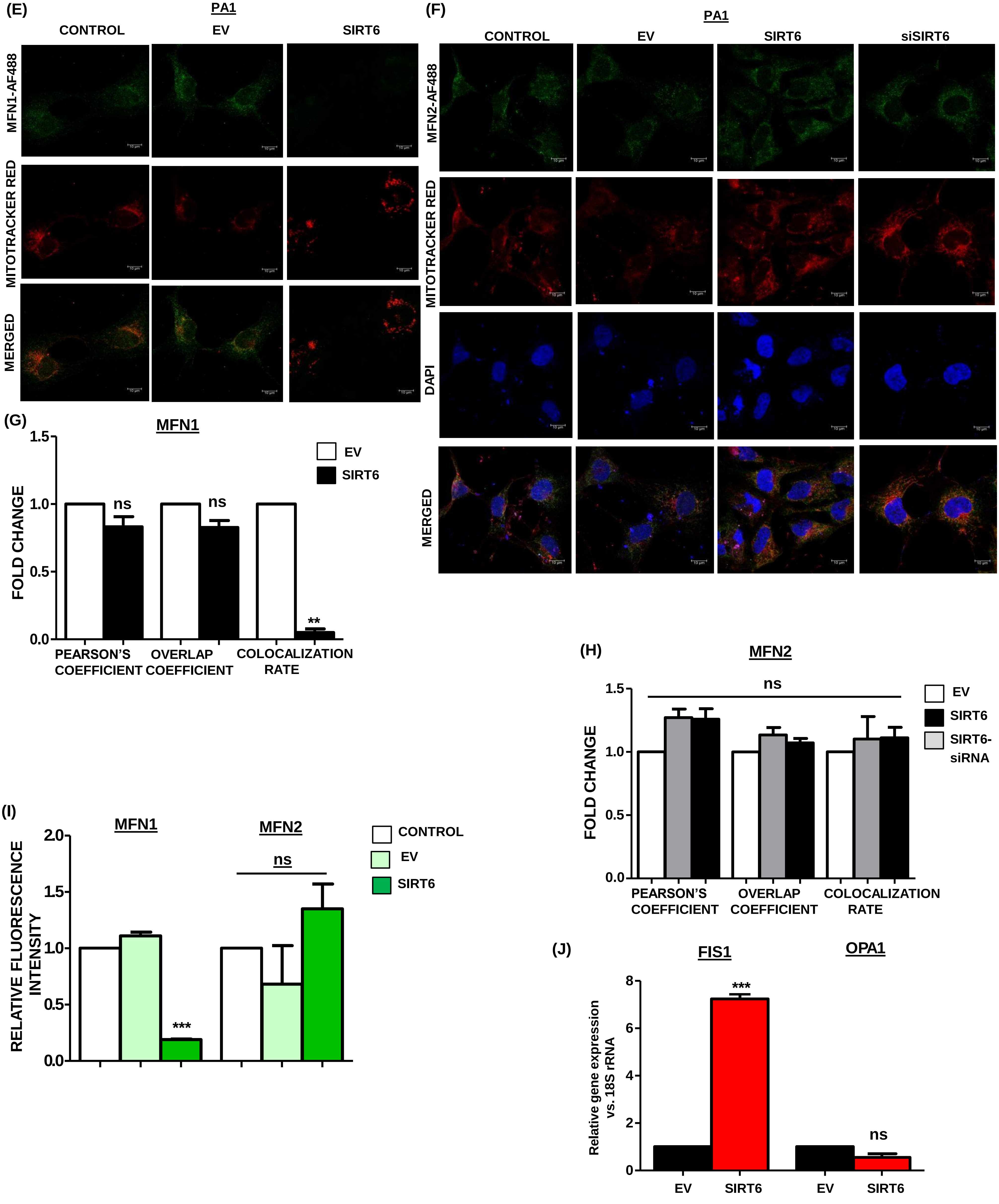
(E)
PA1
CONTROL
EV
SIRT6
MFN1-AF488
MITOTRACKER RED
MERGED
(F)
PA1
CONTROL
EV
SIRT6
siSIRT6
MFN2-AF488
MITOTRACKER RED
DAPI
MERGED
(G)
MFN1
EV
SIRT6
FOLD CHANGE
1.5
1.0
0.5
0.0
ns
ns
**
PEARSON'S COEFFICIENT
OVERLAP COEFFICIENT
COLOCALIZATION RATE
(H)
MFN2
ns
EV
SIRT6
SIRT6-siRNA
FOLD CHANGE
1.5
1.0
0.5
0.0
PEARSON'S COEFFICIENT
OVERLAP COEFFICIENT
COLOCALIZATION RATE
(I)
MFN1
MFN2
CONTROL
EV
SIRT6
RELATIVE FLUORESCENCE INTENSITY
2.0
1.5
1.0
0.5
0.0
ns
***
(J)
FIS1
OPA1
Relative gene expression vs. 18S rRNA
8
6
4
2
0
***
ns
EV
SIRT6
EV
SIRT6

Supplementary Figure 3: (A) qPCR analysis showed increased HIF1α mRNA levels in EV-transfected and SIRT6-transfected PA1 cells (n=3). (B) p-DRP1^{ser616} expression w.r.t total-DRP1 was checked in the presence of HIF1α-siRNA through western blot studies. (C) Confocal microscopy staining with Mitotracker Red and p-DRP1^{ser616}-AF488 in EV, SIRT6 and SIRT6-siRNA transfected PA1 cells. n=3, Scale bar=10 µm. (D) Calculation of colocalization between Mitotracker Red and p-DRP1^{ser616}-AF488 in EV, SIRT6 and SIRT6-siRNA transfected PA1 cells. Mean fluorescent intensity (fold change) of p-DRP1^{ser616}-AF488. (E) Confocal microscopy staining with Mitotracker Red and MFN1-AF488 in Control (untransfected), EV, SIRT6 and SIRT6-siRNA Transfected PA1 cells. n=3, Scale bar=10 µm. (F) Calculation of colocalization between Mitotracker Red and MFN1-AF488 in EV, SIRT6 and SIRT6-siRNA transfected PA1 cells. (G) Confocal microscopy staining with Mitotracker Red and MFN2-AF488 in Control (untransfected), EV, SIRT6 and SIRT6-siRNA transfected PA1 cells. n=3, Scale bar=10 µm. (H) Calculation of colocalization between Mitotracker Red and MFN2-AF488 in EV, SIRT6 and SIRT6-siRNA transfected PA1 cells. (I) Mean Fluorescent Intensity (fold change) between Control (untransfected), EV-transfected and SIRT6-transfected for MFN1-AF488 and MFN2-AF488 SIRT6-transfected PA1 cells (n=3). (J) qPCR analysis to check mRNA levels of FIS1 and OPA1 in EV vs. SIRT6-transfected PA1 cells. n=3, Paired two-tailed t-test was done to calculate the P-value. Error bars represent standard error of mean (SEM) from three independent experiments. * (P-value<0.05), ** (P-value<0.01), *** (P-value<0.001).

SUPPLEMENTARY FIGURE 4

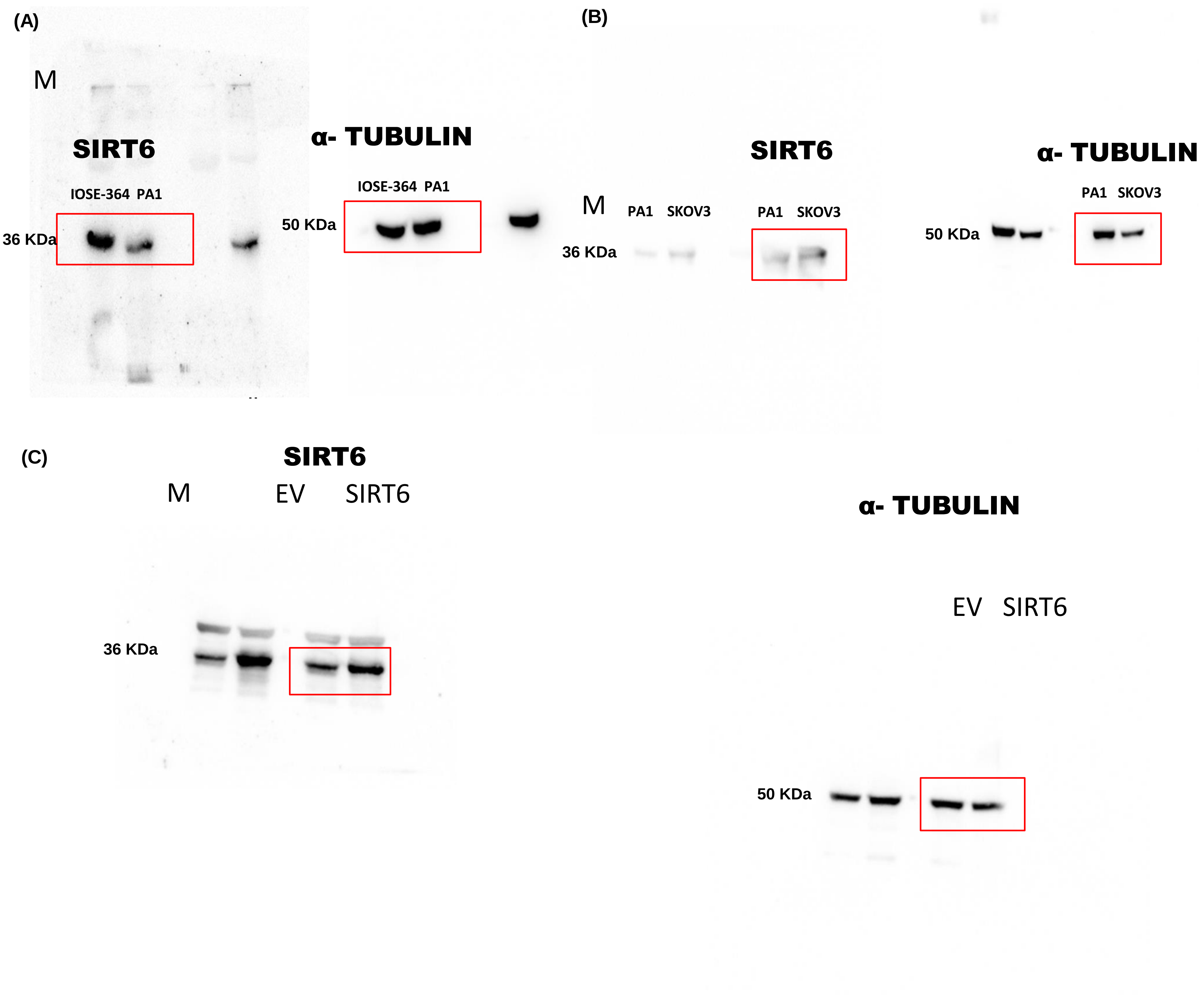

Supplementary Figure 4: (A,B) Full blot of Figure 1D. (C) Full blot of Supplementary Figure 1C.

SUPPLEMENTARY FIGURE 5

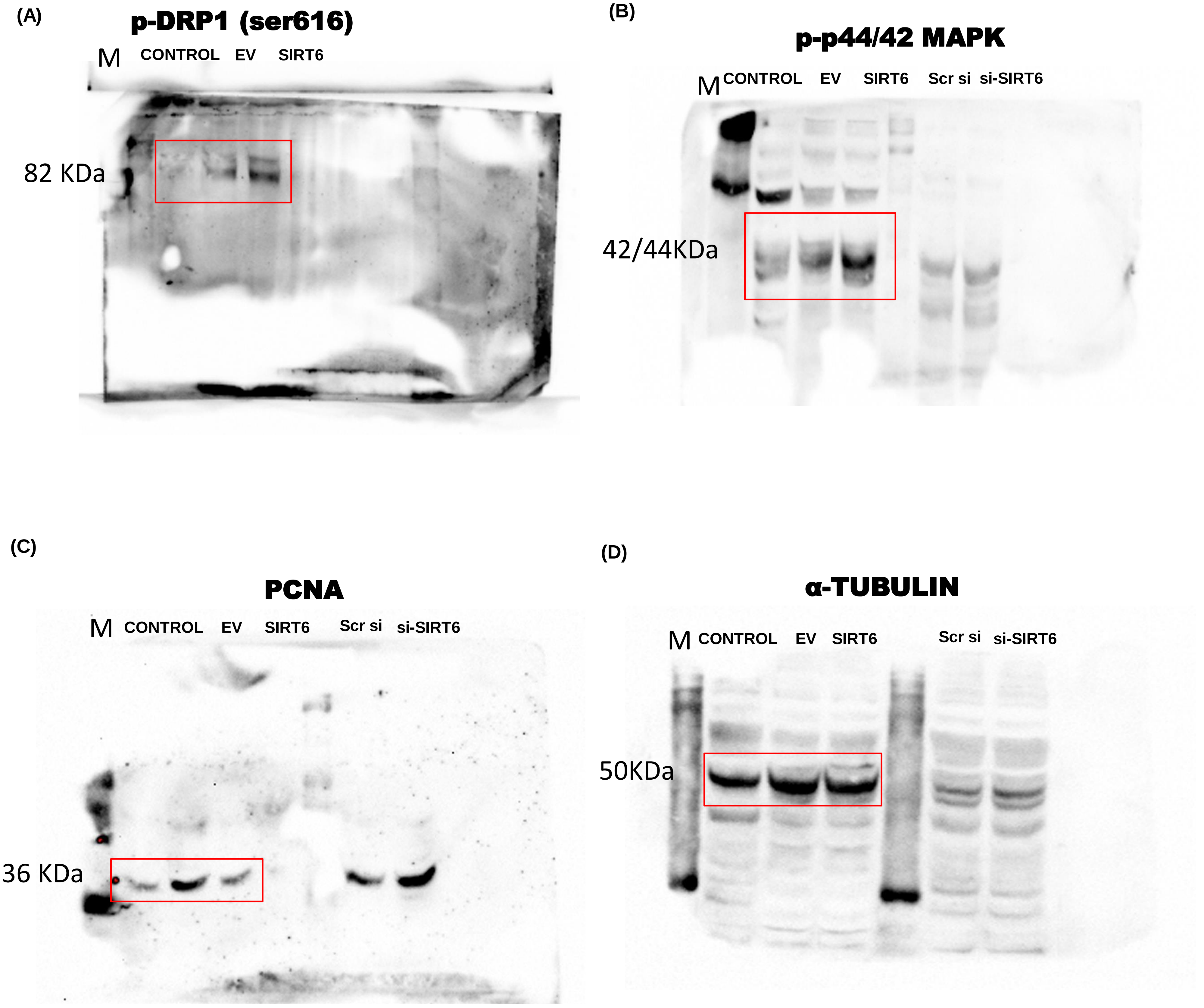

SUPPLEMENTARY FIGURE 5

(E)

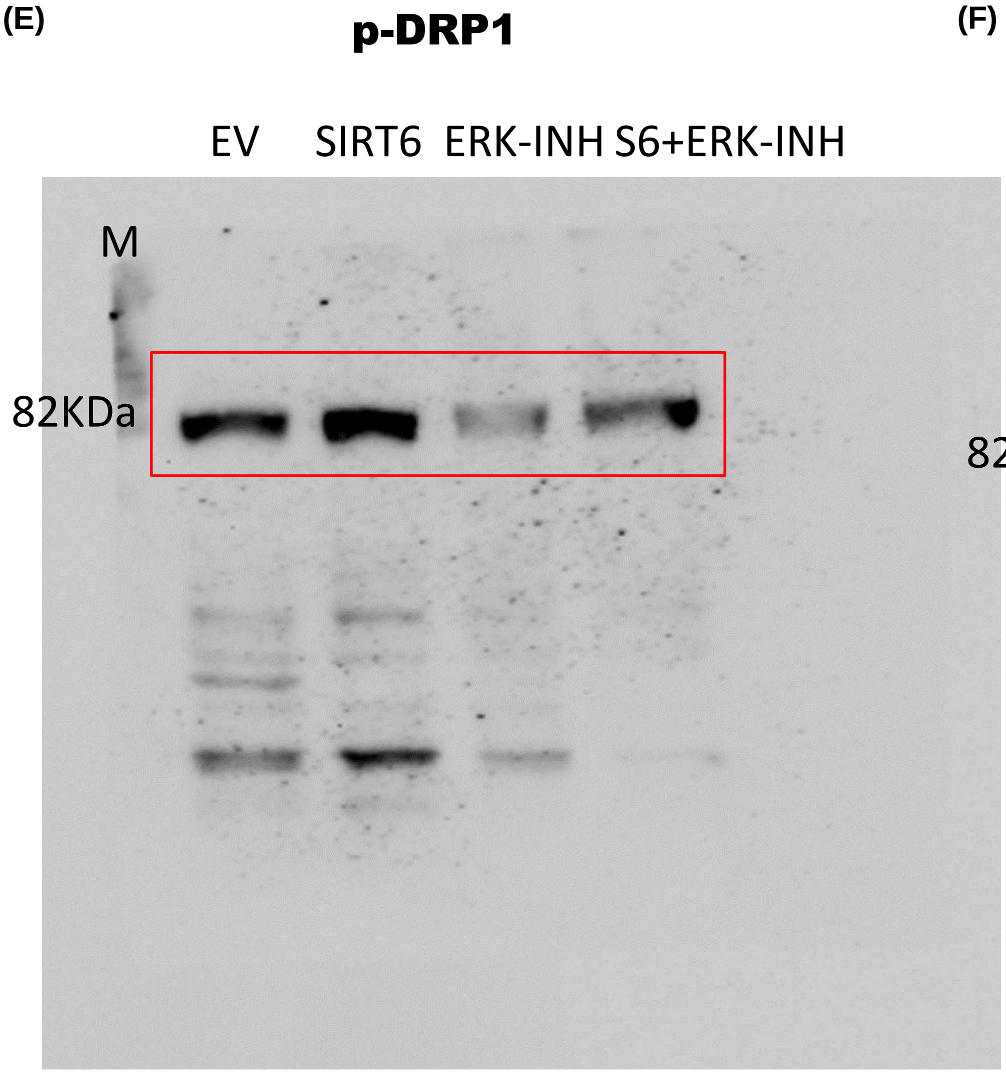

(F)

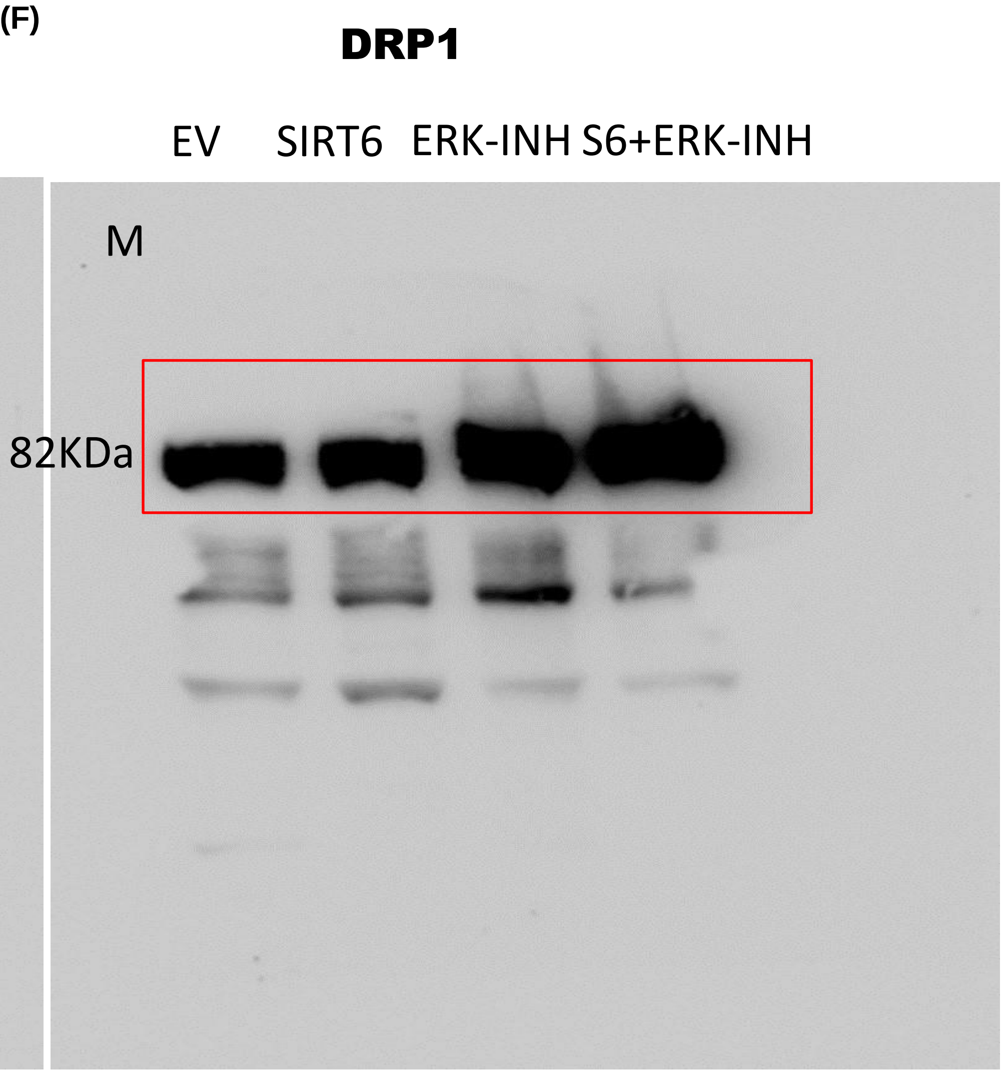

(G)

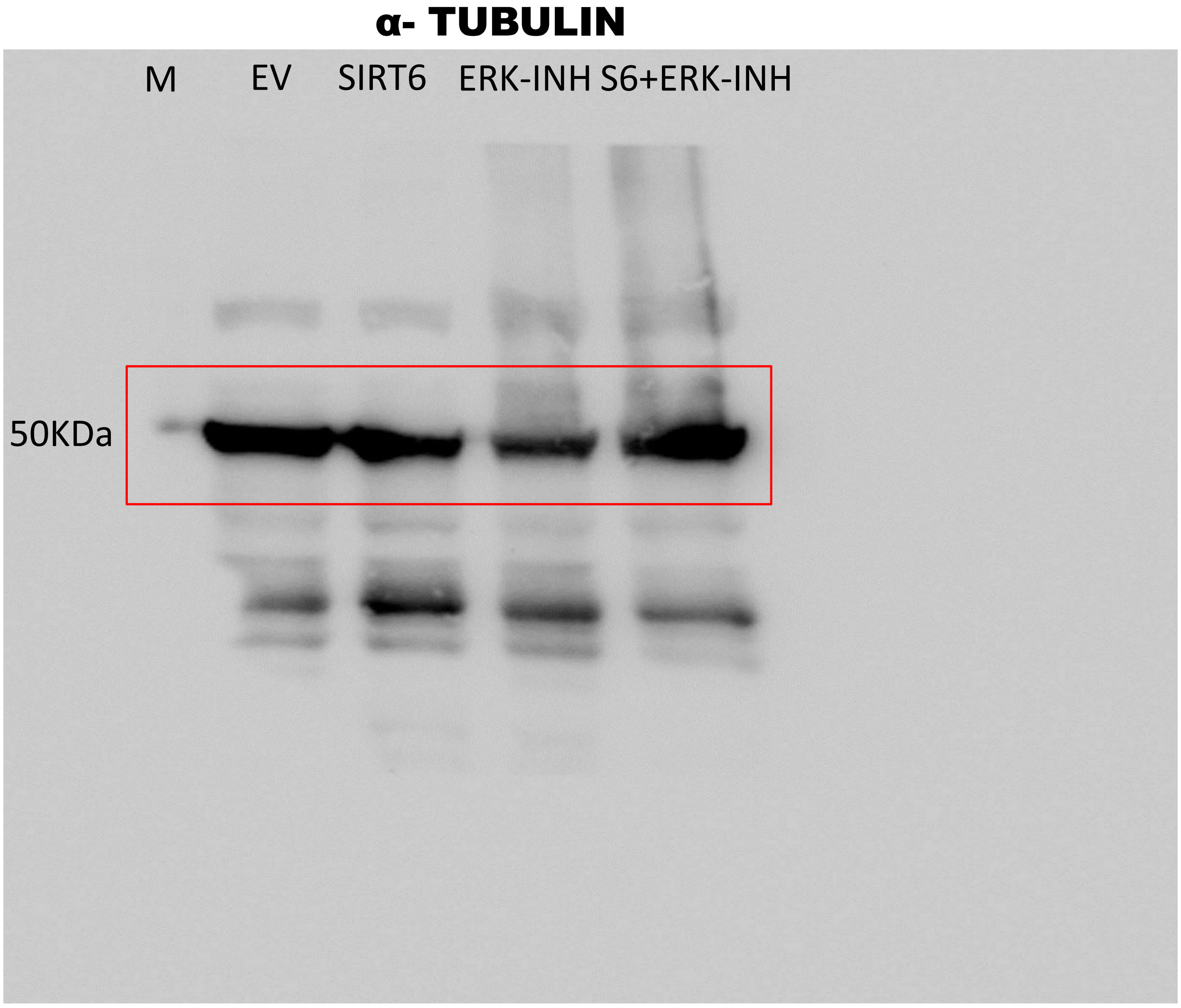

Supplementary Figure 5: (A-D) Full blots of figure 4B. **(E-G)** Full blot of figure 4C

SUPPLEMENTARY FIGURE 6

(A) (B)

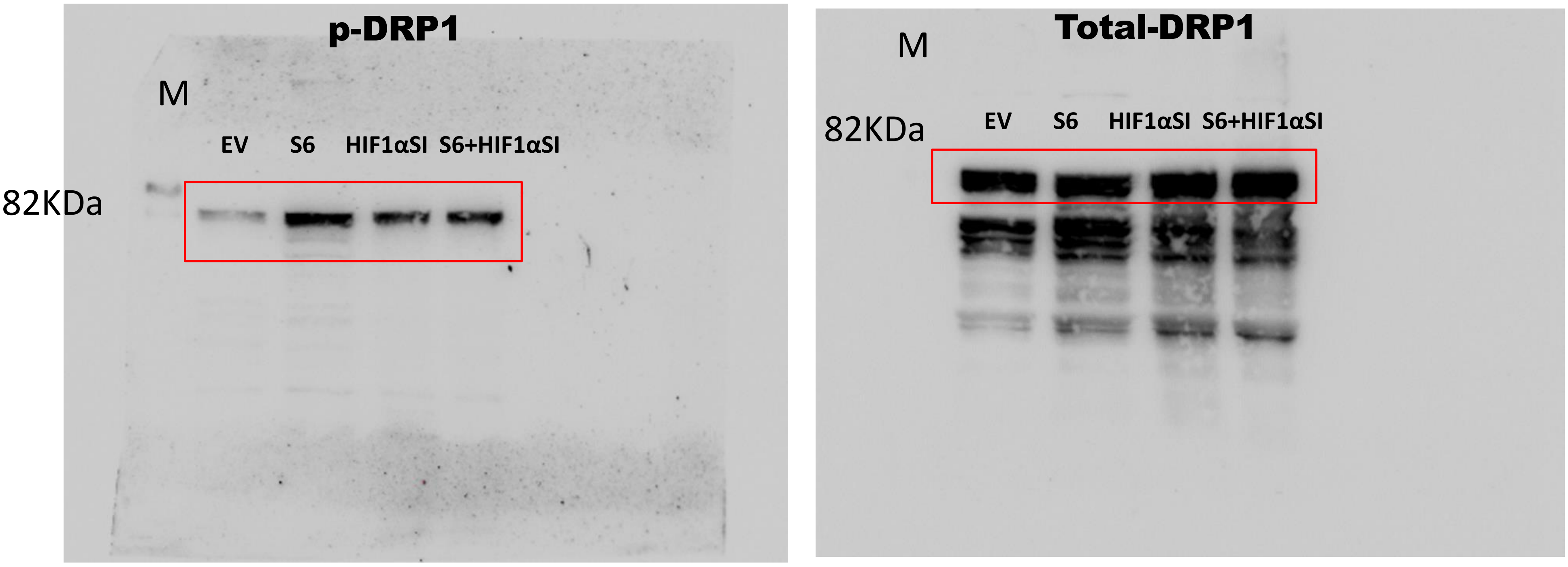

Supplementary Figure 6: (A,B) Full blot of Supplementary Figure 3B.
